# A User-Friendly Kinetic Model Incorporating Regression Models for Estimating Pesticide Accumulation in Diverse Earthworm Species Across Varied Soils

**DOI:** 10.1021/acs.est.4c06642

**Published:** 2024-07-31

**Authors:** Jun Li, Mark E. Hodson, Colin D. Brown, Melanie J. Bottoms, Roman Ashauer, Tania Alvarez

**Affiliations:** † Department of Environment and Geography, University of York, York YO10 5NG, U.K.; ‡ Jealotts Hill International Research Centre, Syngenta Ltd, Warfield, Bracknell RG42 6EY, U.K.; § Syngenta Crop Protection AG, Rosentalstr. 67, 4058 Basel, Switzerland

**Keywords:** uptake, elimination, dermal, gut, lipid, porewater, risk assessment

## Abstract

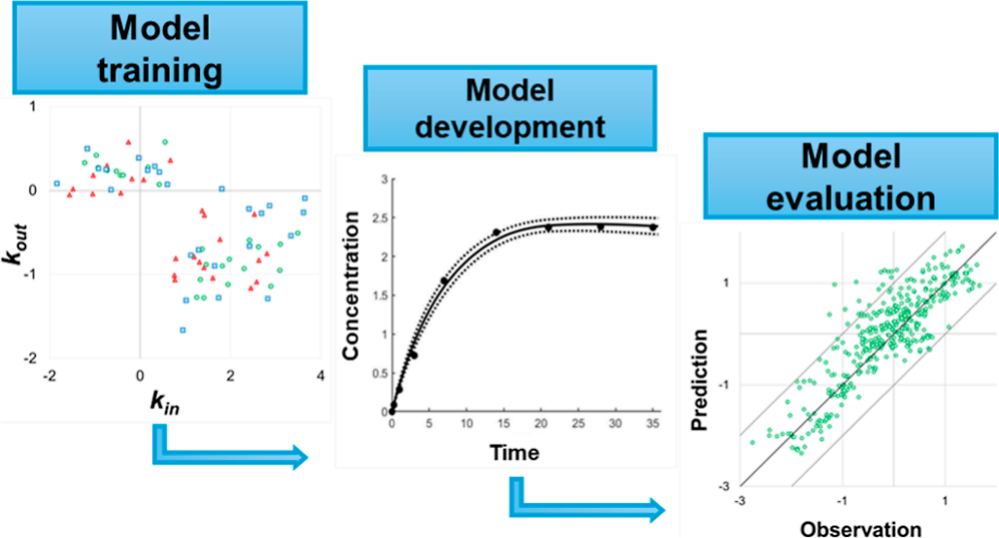

Existing models for estimating pesticide bioconcentration in earthworms exhibit limited applicability across different chemicals, soils and species which restricts their potential as an alternative, intermediate tier for risk assessment. We used experimental data from uptake and elimination studies using three earthworm species (*Lumbricus terrestris*, *Aporrectodea caliginosa*, *Eisenia fetida*), five pesticides (log *K*_ow_ 1.69–6.63) and five soils (organic matter content = 0.972–39.9 wt %) to produce a first-order kinetic accumulation model. Model applicability was evaluated against a data set of 402 internal earthworm concentrations reported from the literature including chemical and soil properties outside the data range used to produce the model. Our models accurately predict body load using either porewater or bulk soil concentrations, with at least 93.5 and 84.3% of body load predictions within a factor of 10 and 5 of corresponding observed values, respectively. This suggests that there is no need to distinguish between porewater and soil exposure routes or to consider different uptake and elimination pathways when predicting earthworm bioconcentration. Our new model not only outperformed existing models in characterizing earthworm exposure to pesticides in soil, but it could also be integrated with models that account for earthworm movement and fluctuating soil pesticide concentrations due to degradation and transport.

## Abstract 2

Our new first-order kinetic models that use predicted uptake and elimination rate constants accurately predict earthworm bioconcentration across contrasting pesticides, soils, earthworm species and various exposure times.

## 1 Introduction

Risk assessment of pesticide residues in the soil environment is of significant interest due to the widespread use of pesticides and their potential effects on terrestrial ecosystems.^1−3^ The assessment of pesticide toxicity to earthworms via laboratory studies (lower-tier option) or site-specific field studies (higher-tier option) is a standard procedure in current regulatory guidance for risk assessment of pesticides.^4^ However, laboratory studies fail to fully reflect ecologically realistic conditions due to their standardization, while field studies are expensive, time-consuming and challenging to extrapolate across diverse agricultural environments.^5^ A modeling approach for estimating uptake of chemicals into earthworms that can link into population-effect models could serve as an intermediate tier for risk refinement that would offer a cost- and time-effective alternative to field testing and minimize animal testing requirements.

Exposure to pesticides in the soil can occur after their application to crops or soils, and the concentration of pesticides can fluctuate significantly over time due to factors such as degradation, rainfall, or repeated applications.^2,6,7^ Kinetic bioconcentration models can be used to estimate the body residues of organic compounds in earthworms at any given time under different exposure scenarios such as constant, fluctuating, or repeated pulsed exposures.^8^ In contrast equilibrium partitioning (EP) models estimate body residues when they are in equilibrium with the compound of interest.^9,10^ Both types of model can be implemented in risk assessments to characterize bioconcentration in earthworms and thereby determine the risk to vertebrates from secondary poisoning.^9^ However, because EP models assume an equilibrium between chemicals in the soil and the exposed organism, they are not suited to exposure scenarios where organisms move and are exposed to varying concentrations of chemical as will be the case for earthworms and pesticides, whereas kinetic models are. In addition, kinetic models can be linked to toxicodynamic models to simulate and predict toxic effects of time-varying exposures on earthworms, thereby providing an option for refining the current risk assessment for in-soil organisms.^7,11^

We identified three previously developed kinetic bioconcentration models that simulate chemical uptake and elimination over time by earthworms in soil.^12,13^ Jager et al.^14^ and Jager^15^ reported similar closed mass balance models incorporating dermal and intestinal uptake routes to estimate the total body residues of the earthworm *Eisenia andrei* resulting from chemical exposure via contaminated soils and/or food. The Armitage and Gobas^16^ model quantifies kinetic uptake of contaminants from air, porewater, soil, and food by earthworms, and their elimination through respiratory exchange, fecal and urinary pathways, reproduction, and metabolic transformation. Our previous evaluations^12,13^ identified Jager et al.^14^ as the best performing model, but with limited applicability across earthworm species and to soils with low (<2%) organic carbon content. All three existing kinetic models require a large number of rate constants for uptake and elimination of chemicals by earthworms as input parameters. These vary greatly across different chemicals, soils, and earthworm species,^17−19^ and need to be calibrated prior to predictive use, further limiting model applicability.

This study aimed to develop an improved kinetic model for estimating the bioconcentration of pesticides by earthworms. A large experimental data set was used to develop relationships to predict earthworm uptake and elimination rate constants based on chemical, soil, and earthworm properties. These rate constants were incorporated into a first-order kinetic model that was evaluated against a large, independent data set obtained from the literature and against the performance of existing models.

## 2 Materials and Methods

### 2.1 Generating Predictive Models for *k*_in_ and *k*_out_

Initially, we used our previous experimental data to generate predictive equations for *k*_in_ and *k*_out_. Li et al.^13^ determined values of uptake and elimination rate constants from experimental data for five pesticides (lenacil, flutriafol, dieldrin, hexachlorobenzene and *p*,*p*′-DDT), five soils and three earthworm species (*Lumbricus terrestris*, *Eisenia fetida*, and *Aporrectodea caliginosa*) following standardized OECD 317 guidelines^20^ and under consistent experimental conditions. This generated 75 uptake (*k*_in_) and elimination (*k*_out_) rate constants each for adult earthworms for both soil porewater and bulk soil concentrations (Table S1). The uptake rate constants calculated based on porewater or bulk soil concentrations ranged from 0.02 to 4392 L porewater kg^–1^ earthworm d^–1^ and 0.02 to 2.30 kg soil kg^–1^ earthworm d^–1^, respectively. *k*_out_, elimination rate constants calculated based on porewater or bulk soil concentrations, ranged from 0.02 to 3.84 d^–1^ and 0.02 to 4.40 d^–1^. The five pesticides are relatively persistent in soil and are not rapidly biotransformed by earthworms (Pesticide Property Database; https://sitem.herts.ac.uk/aeru/ppdb/). Log *K*_ow_ (octanol–water partition coefficient) ranged from 1.69 to 6.63, log *K*_om_ [measured sorption coefficient (*K*_d_, the ratio of chemical concentration in soil to concentration in porewater) normalized to soil organic matter (OM)] ranged from 1.22 to 5.23 and TPSA (fragment-based polar surface area from N, O, S, P polar coefficients) ranged from 0 to 50.9 Å^2^. Soil OM content ranged from 0.97 to 39.9%, clay content (clay) from 4.02 to 50.0%, cation exchange capacity (CEC) from 1.41 to 88.8 cmol+/kg and pH from 5.11 to 6.97. The three earthworm species had lipid contents (Lipid) ranging from 1.55 to 2.64% (wet weight) and specific surface areas (SSA) ranging from 0.70 to 1.45 m^2^ kg^–1^.

Stepwise multiple-linear regression analysis in SPSS (version 25.0) was used to develop models for estimating *k*_in_ and *k*_out_ based on readily measurable pesticide, soil, and earthworm properties using the data reported in Li et al.^13^ Chemical properties included were log *K*_d_, log *K*_om_, log *K*_ow_ and TPSA. Soil properties were OM, clay, CEC, and pH. Earthworm properties were lipid, SSA, and SSAlipid (i.e., SSA multiplied by lipid). Intercorrelation between chemical, soil, and earthworm descriptors was analyzed using Pearson statistical bivariate correlation analyses in SPSS. Significant intercorrelation (*p* < 0.01) existed between chemical descriptors (log *K*_ow_, log *K*_om_, and TPSA), soil descriptors (OM, clay, CEC, and pH), and earthworm descriptors (lipid, SSA, and SSAlipid). Only one descriptor (that resulted in the highest *R*^2^ value of the resultant regression) from each intercorrelated group was included in the regression analysis to avoid multicollinearity (variance inflation factor < 2). The best model was identified based on the determination coefficient (*R*^2^), the adjusted determination coefficient (adjusted *R*^2^), and the root-mean-square error (RMSE) when all the descriptors in the model were significant at 95% confidence level. The robustness and reliability of the developed models were assessed internally using the leave-one-out cross-validated correlation coefficient (*Q*_LOO_^2^) and the leave-one-out cross-validated concordance correlation coefficient (CCC), using caret and DescTools package, respectively, in R software (R version 3.5.1). The calculation of RMSE, *Q*_LOO_^2^ and CCC are provided in the Supporting Information.

### 2.2 Testing the *k*_in_ and *k*_out_ Models with a First-Order Kinetic Model against Literature Data

We incorporated the *k*_in_ and *k*_out_ relationships that we derived into a first-order kinetic model (eq 1) of the type frequently used to describe uptake and elimination of pesticides in earthworms.^17,18,20,21^
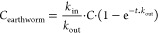
1where *C*_earthworm_ is the concentration of substance in the earthworm (mg kg^–1^ wet weight); *C* is the concentration of substance in either the porewater (mg L^–1^) or bulk soil (mg kg^–1^ dry weight); *k*_in_ is the uptake constant for tissue related to either porewater (L porewater kg^–1^ earthworm d^–1^) or soil (kg soil kg^–1^ earthworm d^–1^) concentrations, *k*_out_ is the elimination rate constant (d^–1^); *t* is time since initial exposure (d).

The models we developed are specifically for adult earthworms, as the training data were derived from adult specimens. Since growth dilution was not observed in the training data reported by Li et al.,^13^ this component is not included in the model. To evaluate the predictive performance of this approach we compiled a large independent data set from the literature, based on all available data published between 2002 and 2022. Details of search terms used are provided in the Supporting Information (Section S1.2). The data set comprised 21 studies providing 402 internal earthworm concentration data points, of which 130 are situated slightly outside the applicability domain of the developed models (Table S5). The concentrations range from 0.002 to 46.9 mg kg^–1^ wet weight, collected from accumulation time series of up to 42 days for 23 organic chemicals, including four ionizable compounds and 19 nonionizable compounds (Table S2). 84 data points were measured between Day 1 and Day 6 of exposure, 242 data points between Day 7 and Day 20, and 76 data points between Day 21 and Day 42. The evaluation data cover four earthworm species (*E. fetida* and *E. andrei*: 360 data points, *A. caliginosa*: 21 data points, and *L. terrestris*: 21 data points). Of the 402 data points, 217 represented either steady state concentrations or a maximum concentration at the end of the exposure period. Log *K*_ow_ values of the compounds in the data set range from 1.99 to 8.19; soil OM contents range from 0.31 to 34.7%.

One parameter included in our regression for predicting *k*_in_ was *K*_om_, the ratio of the pesticide concentration in porewater to bulk soil normalized to the soil OM. Applying these relationships to the independent test data required a porewater concentration. However, porewater concentrations were only measured in Carter et al.^21^ providing 24 data points across 4 compounds (Table S2). When none was available, we calculated a value for the porewater concentration using eq 2.
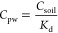
2

In these cases, the value of *K*_d_ used was determined from a relationship derived by a stepwise multiple-linear regression analysis on data from our experimental study.^13^ This study includes 75 sorption coefficients (*K*_d_) determined for the five pesticides, five soils, and three earthworm species treatments (Table S1); the different earthworm treatments used different temperatures and quantities of soil. Chemical (log *K*_ow_, and TPSA) and soil (OM, CEC, and pH) properties were initially included in the regression. The regression analysis suggested use of a combination of the chemical’s hydrophobicity (log *K*_ow_) and the soil OM content to predict the *K*_d_ of neutral pesticides. We extrapolated the model to monovalent ionizable organic compounds by replacing log *K*_ow_ with the pH-adjusted octanol–water partition coefficient (log *D*_ow_, which has the same value as log *K*_ow_ for neutral compounds). The model equation is given below
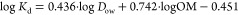
3With *N* = 75, *R*^2^ = 0.591, adjusted *R*^2^ = 0.580 (*p* < 0.05), RMSE = 0.749, *Q*_LOO_^2^ = 0.557, CCC = 0.722.

The applicability domain of the model parameters log *D*_ow_ and OM ranges from 1.69 to 6.63 and 0.97 to 39.9%, respectively. When the independent published studies that we used to test our model reported porewater or bulk soil concentrations at different time points (19 of the 21 studies, Table S2), we fitted a first-order kinetic model to the data to describe the degradation kinetics (eq 4) following the OECD Guideline 317.^20^ The other two studies reported that initial concentrations did not decrease over time.
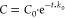
4where *C*_0_ is the initial concentration of substance in porewater (mg L^–1^) or bulk soil (mg kg^–1^ dry weight), and *k*_0_ is the degradation rate constant (d^–1^) calculated based on chemical concentrations in porewater or bulk soil.

Substituting this value of *C* into eq 1, gave eq 5(20)
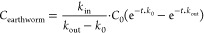
5

All kinetic models were implemented using the ODE solver in Matlab (R2021b) with the BYOM modeling platform (version 6.0) (http://debtox.info/byom.html). The ordinary differential equations for eqs 1 and 4 are provided in the Supporting Information.

### 2.3 Evaluation and Comparison of the Developed Model with Existing Models

We compared the performance of our newly developed model to that of an existing EP model^22^ and a kinetic model,^14^ which we had previously found to be the best performing of existing models for estimating bioconcentration of organic compounds in earthworms.^12^ The EP model of Belfroid et al.^22^ calculates steady-state internal concentration of earthworms as the sum of chemical uptake from interstitial water, via passive diffusion as determined by a bioconcentration factor, and soil ingestion as determined by soil uptake rate (estimated by feeding rate multiplied by uptake efficiency) and the total elimination rate. Its performance was evaluated using a subset of our independent data taken from the literature (Table S3) comprising 217 data points representing steady-state or maximum internal concentrations in earthworms. The data were taken from 18 studies, with 210 data points for *E. fetida* and *E. andrei*, and only seven data points for *L. terrestris*. Of the total 19 organic compounds in this subset, 15 were nonionic and four were ionic. The compounds ranged from relatively hydrophilic to relatively hydrophobic, with log *K*_ow_ ranging from 1.99 (metalaxyl) to 8.19 (orlistat). The kinetic model of Jager et al.^14^ estimates earthworm internal concentrations over time by calculating net uptake flux across the outer epidermis and gut. The model is parametrized for PCB 153 for a single soil type (OM 10.5%) and earthworm species (*E. Andrei*). Therefore, we used PCB 153 data from our independent data set to evaluate the performance of this model (Table S4). This comprised 44 uptake kinetic data points (internal concentrations as part of a time series) from five studies involving two earthworm species (*E. fetida*, *E. andrei*) and 18 soil types with OM contents ranging from 0.81 to 34.7%. Predictions of both models were compared to those of our newly developed models and assessed through Nash–Sutcliffe Efficiencies (NSE) (see Supporting Information) and the percentage of predictions within a factor of 10, 5, and 3 of the measured values. When NSE equals 1, predicted values match observed values perfectly. As model performance decreases NSE values decrease and become negative.

## 3 Results and Discussion

### 3.1 Regression Models for Predicting *k*_in_ and *k*_out_

In our study, the best model for predicting uptake rate constants (*k*_in_) produced by stepwise regression of our data used a combination of a chemical descriptor (log *K*_om_), a soil descriptor (OM), and an earthworm descriptor (SSAlipid); it explained 96.4 and 73.8% of the variation in the experimentally determined porewater-based and bulk soil-based uptake rate constants, respectively (Table 1).

**Table 1:** Multiple Linear Regression Models for Predicting *k*_in_ and *k*_out_^a^

exposure route	model	*N*	*R*^2^	adjusted *R*^2^	RMSE	*Q*_LOO_^2^	CCC	equation
porewater	*k*_in_	75	0.964	0.962	0.276	0.960	0.979	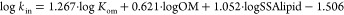
	*k*_out_	75	0.805	0.800	0.258	0.749	0.858	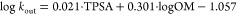
soil	*k*_in_	75	0.738	0.727	0.255	0.709	0.833	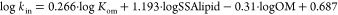
	*k*_out_	75	0.880	0.875	0.234	0.818	0.901	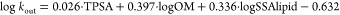

a *k*_in_: uptake rate constant in tissue from porewater (L porewater kg^–1^ earthworm d^–1^) or bulk soil (kg soil kg^–1^ earthworm d^–1^), respectively; *k*_out_: elimination rate constant (d^–1^) calculated based on chemical concentrations in the porewater or bulk soil, respectively.; *N*: number of observations; *R*^2^ and adjusted *R*^2^: determination coefficient and adjusted determination coefficient (*p* < 0.05 for all values), respectively; RMSE: root-mean-square error; *Q*_LOO_^2^: leave-one-out cross-validated correlation coefficient; CCC: leave-one-out cross-validated concordance correlation coefficient; log *K*_om_: measured distribution coefficient normalized by soil OM (1.22–5.23); OM: soil organic matter content (0.97–39.9%); SSAlipid: earthworm specific surface area (0.70–1.45 m^2^ kg^–1^) multiplied by earthworm lipid content (1.55–2.64%). TPSA: fragment-based polar surface area from N, O, S, P polar coefficients (0–50.9 Å^2^). The applicability domain of each model parameter (log *K*_om_, OM, SSA and lipid) is provided in the parentheses. The QSAR Model Reporting Formats (QMRF) for the developed regression models are provided in the Supporting Information.

Log *K*_om_ explained the largest variation in *k*_in_ according to the *R*^2^ change and *F* change (*F*-statistic, see Tables S6 and S8), followed by OM or SSAlipid. Chemical hydrophobicity (log *K*_ow_) has been identified previously as the primary factor driving uptake rates of organic compounds in earthworms.^18,23,24^ In our study, log *K*_om_ had a strong positive correlation with log *K*_ow_ (*r* = 0.72, *p* < 0.01) and a negative correlation with TPSA (*r* = −0.87, *p* < 0.01). Regression analysis showed that log *K*_om_ was superior to log *K*_ow_ and TPSA in capturing the variance in pesticide uptake rate constants of earthworms. In addition to properties of the chemical to which the earthworm was exposed, previous studies indicate that soil OM,^23^ and earthworm properties such as lipid content^19,25^ and SSA^19^ also influence uptake rates of organic compounds by earthworms. Our study is consistent with this, and incorporation of these soil and earthworm descriptors significantly improved model fit.

TPSA and OM were the best predictor combination for porewater-based elimination rate constants (*k*_out_) accounting for 80.5% of variation in values (Table 1). TPSA explained the majority of the variation in porewater-based *k*_out_ (72.3%), slightly outperforming log *K*_ow_ (66.2%). Inclusion of an earthworm property (SSAlipid) as an additional model input slightly improved the model fit for bulk soil-based elimination rate constants (*k*_out_, *R*^2^ = 0.88). The dependence of elimination rate constants on hydrophobicity (log *K*_ow_ or TPSA) is consistent with previous studies,^26−28^ which demonstrated that the strong binding of hydrophobic compounds to earthworm lipids may retard elimination. For example, Matscheko et al.^27^ observed a strong negative correlation between elimination rate constants and log *K*_ow_ (which itself is correlated negatively to TPSA) for polycyclic aromatic hydrocarbons and polychlorinated biphenyls. In addition, it has been reported that OM facilitates elimination of organic compounds by earthworms due to sorption of the compounds to excreted OM.^17,23^ Our results confirm that OM plays a role in determining *k*_out_. Carter et al.^19^ noted that the effect of interspecies differences on porewater-based *k*_out_ is negligible but that the effect of interspecies differences on bulk soil-based *k*_out_ has not yet been fully investigated. Our results also indicate that interspecies differences had a negligible effect on porewater-based *k*_out_, whereas they had a slight but significant impact on bulk soil-based *k*_out_.

As shown in Table 1, all the developed models demonstrate high robustness (*Q*_LOO_^2^ > 0.709) and reliability (CCC > 0.833) based on internal cross-validation. The developed models were better able to predict *k*_in_ from soil porewater concentrations than bulk soil concentrations, whereas the reverse was true for *k*_out_. Although our models were developed using both measured porewater and bulk soil concentrations, almost all the independent data used to test the models only reported bulk soil concentrations; we predicted pore water concentrations using eq 2. This may, in part explain the similarity in accuracy of prediction against the independent evaluation data. Because of this similarity, only the results of model evaluation based on predicted porewater concentrations are presented in the main manuscript. Additional model evaluation results based on bulk soil concentrations are provided in the Supporting Information.

### 3.2 Evaluation of Developed Kinetic Models against Independent Data

**Figure 1:**
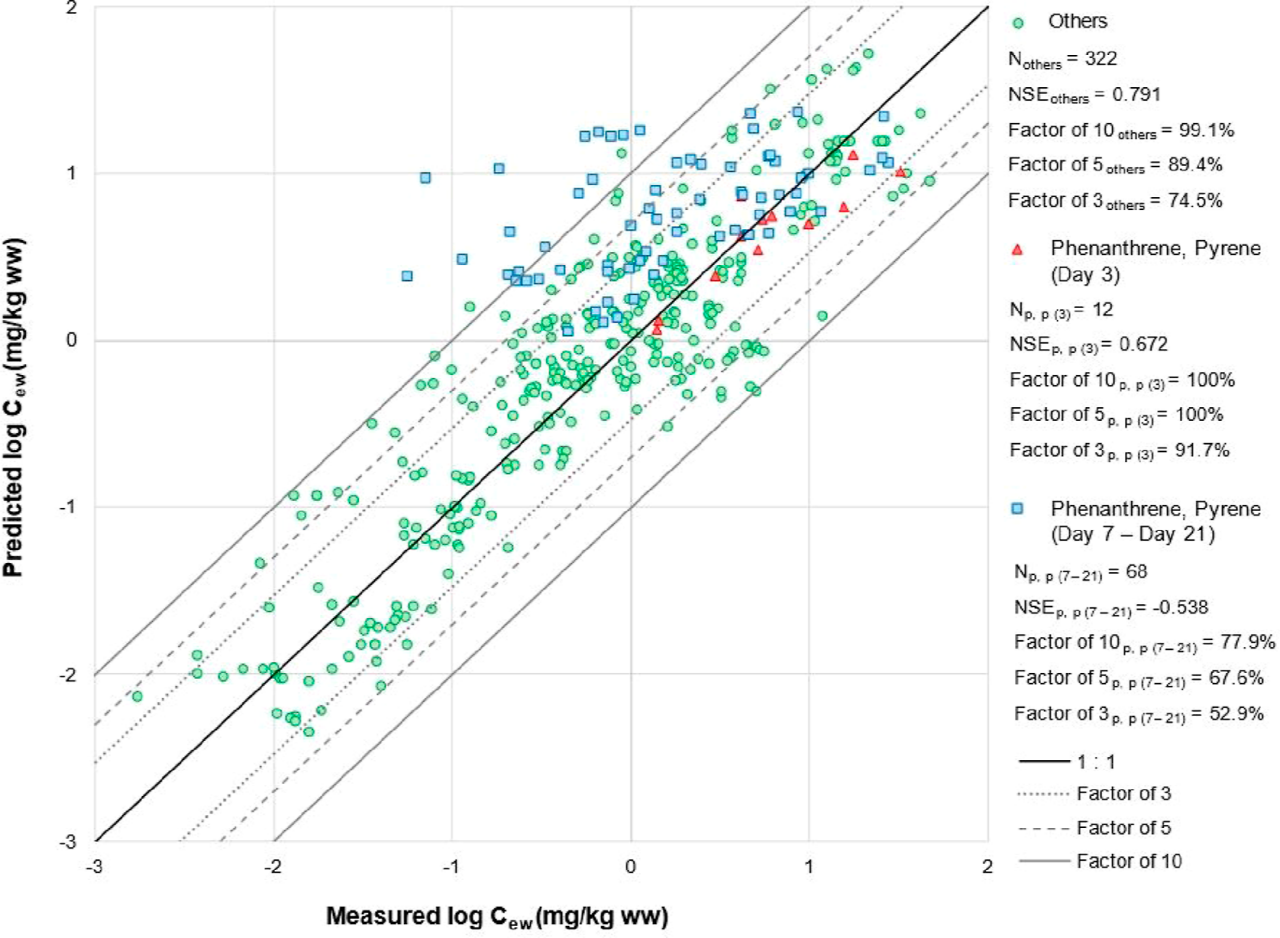
Evaluation of the predictive performance of our new kinetic model based on porewater concentrations against the independent data for phenanthrene, pyrene, and other organic compounds. “Others” (in green) is the full evaluation data set excluding data for phenanthrene and pyrene. “p,p (3)” and “p,p (7–21)” and “phenanthrene, pyrene (Day 3)” and “phenanthrene, pyrene (Day 7–Day 21)” are the evaluation data sets for phenanthrene and pyrene measured in the uptake phase at Day 3 (red triangles) and for Days 7 to 21 (blue squares), respectively. The central black solid line represents a perfect model fit (1:1 line). The gray dotted, gray dashed, and outer solid lines represent a 3-fold, 5-fold, and 10-fold difference between the predicted and observed values, respectively.

Although the evaluation data set covers a wider range of chemicals (both nonionizable and ionizable), soils, and earthworm species than the data used to produce our new models, the newly derived models worked well; they predicted internal concentrations of the majority of organic compounds in earthworms based on both porewater and bulk soil concentrations with an overall NSE of 0.690 and 0.643, respectively. Over 95.5, 86.1, and 71.4% of body load predictions based on porewater concentrations were within a factor of 10, 5, and 3 of the corresponding observed values, respectively (Figure 1). Predictions based on bulk soil concentrations were also good, with more than 93.5, 84.3, and 67.2% of predictions being within a factor of 10, 5, and 3 of the corresponding observed values, respectively (Figure S1). Further model evaluation used subsets of our independent data set to consider model performance for nonionizable versus ionizable compounds, phenanthrene and pyrene, for different exposure times, and for different earthworm species. Performance for nonionizable and ionizable compounds was similar and is presented in Figure S6. Performance for data points falling inside the applicability domain of the developed models (NSE > 0.706) is slightly better than for data points outside the applicability domain of the model (NSE > 0.440) and is presented in Figure S7. The performance of the model for the other subsets is presented and discussed below.

#### 3.2.1 Phenanthrene and Pyrene Predictions

The developed models produced accurate predictions for 21 of the organic compounds in the evaluation data set; these are compounds for which earthworm internal concentrations either reached a steady state or decreased slightly after 21 days of exposure. In contrast, the models performed slightly less well for phenanthrene and pyrene. Earthworm internal concentration of these compounds reached a peak within the first few days of exposure followed by a substantial decline after about 7 days despite the exposure experiment still being in the uptake phase. This could be due to biotransformation of the compound, the initiation of active excretion mechanisms or increased adsorption of the compound to the soil reducing bioavailability.^29−32^ Any of these processes would result in a lower internal concentration of parent compounds than the model predicted.

The model performed well during the early uptake phase, Day 3, with 91.7% of predictions (*N* = 12) within a factor of 3 of the measured values (Figure 1). However, the model tended to overestimate internal concentrations from Days 7 to 21 of the uptake phase, with only 52.9% (*N* = 68) of predictions falling within a factor of 3 of the corresponding measured values and the fit of predictions to the measured values having a negative NSE value (Figure 1). Phenanthrene and pyrene are not the only compounds for which this behavior might be observed. For example, it has been reported that the biotransformation process in earthworms strongly influences the bioconcentration of some pesticides such as endosulfan,^33^ and R-cypermethrin.^34^ Therefore, future model development should take into account reasons for the shape of uptake curves that show decreases in internal concentration during uptake.

#### 3.2.2 Exposure Time

**Figure 2:**
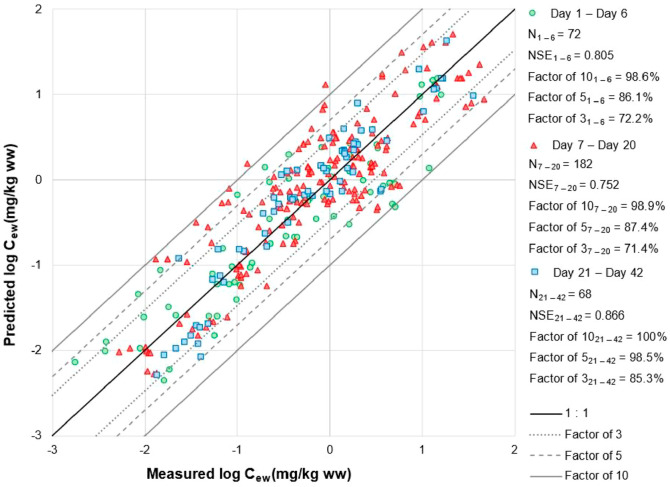
Evaluation of the predictive performance of our new kinetic model based on porewater concentrations against the independent data at different exposure time periods. Data for phenanthrene and pyrene are excluded. “1–6”, “7–20”, and “21–42” and “Day 1–Day 6”, “Day 7–Day 20”, and “Day 21–Day 42” are the subsets of the evaluation data set for uptake from Days 1 to 6 (green circles), Days 7 to 20 (red triangles), and Days 21 to 42 (blue squares), respectively. The central black solid line represents a perfect model fit (1:1 line). The gray dotted, gray dashed, and outer solid lines represent a 3-fold, 5-fold, and 10-fold difference between the predicted and observed values, respectively.

When evaluated against the independent data set, excluding phenanthrene and pyrene, the models developed from both porewater and bulk soil concentrations accurately predicted internal concentrations of organic compounds in earthworms during the early (Day 1 to Day 6), mid (Day 7 to Day 20), and late (Day 21 to Day 42) uptake phases (NSE > 0.707) (Figures 2 and S2). Comparing model performance across different exposure time periods, the developed models provided slightly better predictions in the late-uptake phase than in mid- and early uptake phases according to the percentage of predictions within a factor of 3 and 5 of the measured values (Figure 2). This suggests that our models were slightly better able to capture variation in the bioconcentration of organic compounds once a steady state was reached compared to during the rapid accumulation phase early in uptake experiments. Moreover, even though experimental concentrations in porewater changed over time due to degradation, this had no significant effect on the performance of the developed models when corrected for using eq 4.

#### 3.2.3 Earthworm Species

**Figure 3:**
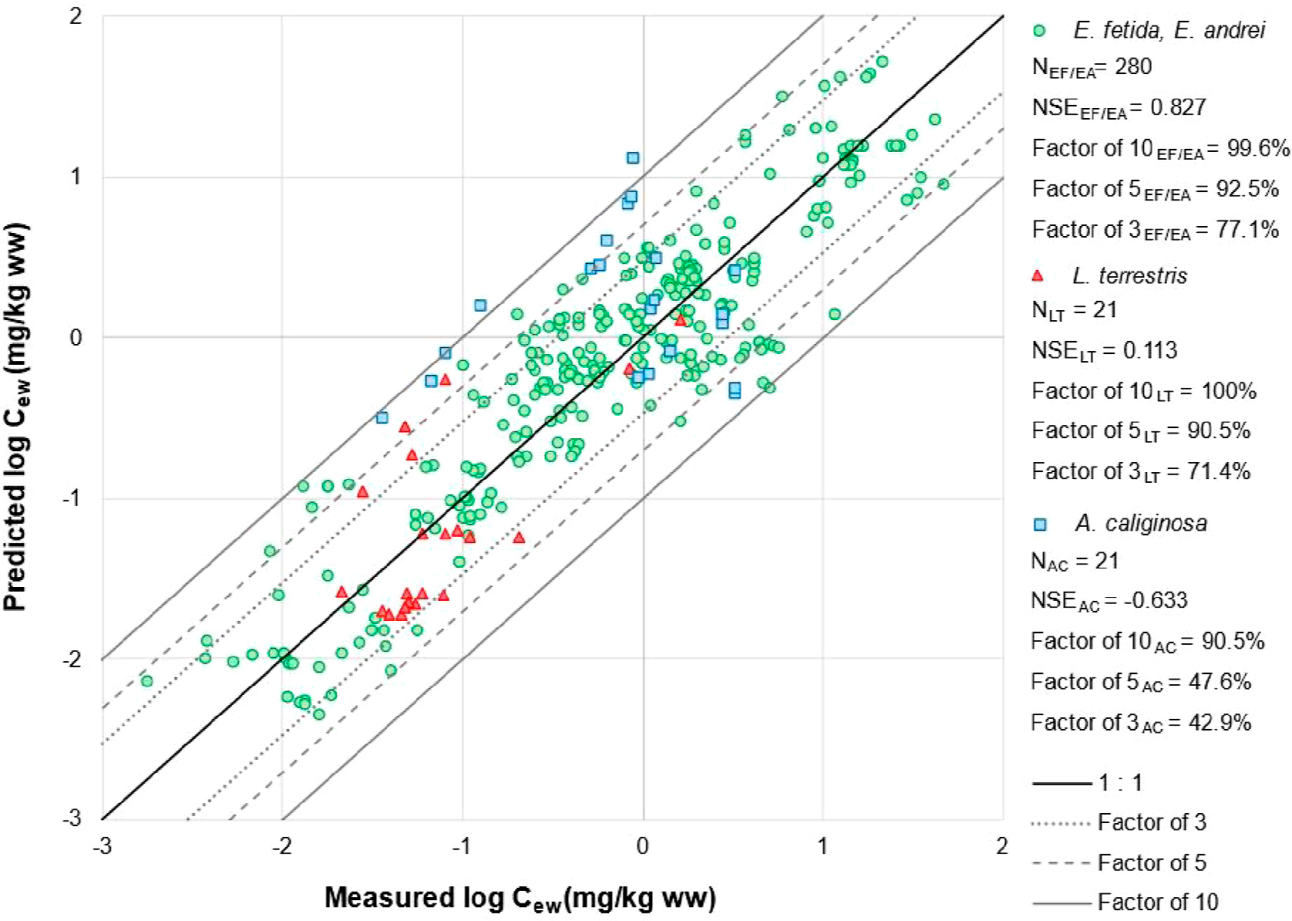
Evaluation of the predictive performance of our new kinetic model based on porewater concentrations against the independent data for different earthworm species. Data for phenanthrene and pyrene are excluded. “EF/EA” (green circles), “LT” (red triangles), and “AC” (blue squares) represent data for the earthworm species *E. fetida*, *E. andrei*, *L. terrestris*, and *A. caliginosa*, respectively. The central black solid line represents a perfect model fit (1:1 line). The gray dotted, gray dashed, and outer solid lines represent a 3-fold, 5-fold, and 10-fold difference between the predicted and observed values, respectively.

Existing studies indicate that interspecies differences in physiology, metabolism, and ecology could significantly influence uptake kinetics of organic compounds in earthworms.^19,25^ However, our previous study demonstrated that physiological characteristics of earthworms, i.e. lipid content and SSA, are the predominant factors explaining interspecies variation in uptake of pesticides.^13^ The developed models incorporate both of these earthworm properties (SSAlipid), and achieved a good prediction of bioconcentration of pesticides for all four earthworm species, with at least 90.5% of predictions falling within a factor of 10 of measured values (Figure 3). Comparing model performance across earthworm species, the developed models provided accurate and comparable predictions for *E. fetida*, *E. andrei*, and *L. terrestris*, but slightly less accurate predictions for *A. caliginosa*, as indicated by the percentage of predictions within a factor of 3 and 5 of measured values (Figure 3).

*A. caliginosa* survives drought by establishing aestivation chambers in the topsoil, a physiological adaptation that is not observed in *L. terrestris* or *E. fetida*.^35^ However, aestivation behavior was not observed in the training data reported by Li et al.^13^ In addition, *L. terrestris*, being larger, likely has a lower metabolic rate compared to the smaller *E. fetida* but *A. caliginosa* is a similar size to *E. fetida*.^36,37^ For these reasons, the predictive performance of the developed models is unlikely to be significantly affected by physiological differences in earthworms. The developed models are as simple as possible while showing great potential for generalization to predict bioconcentration across earthworm species, addressing a significant limitation of existing EP and kinetic models.^12,13^ However, the data available in the literature to test our models for *L. terrestris* and *A. caliginosa* are relatively few and show limited variation. A large and high-quality data set on uptake kinetic data for earthworm species other than *E. fetida* and *E. andrei* is required for further evaluation of our models.

### 3.3 Comparisons of Model Performance against the Best-Performing Existing Models

#### 3.3.1 EP Model of Belfroid et al.^22^

In this study, we found that our new porewater-based model (Figure S4A) and bulk soil-based model (Figure S4B) outperformed the best existing EP model by Belfroid et al.^22^ (Figure S4C) in predicting both steady-state concentrations and, when a steady state was not reached in studies, the maximum reported internal concentrations for earthworms during the uptake phase. The porewater-based model achieved the best predictive performance overall. In particular, our new model performed better than that of Belfroid et al.^22^ for larger earthworm species (i.e., *L. terrestris*) and for hydrophilic compounds (i.e., metalaxyl) (all predictions within a factor of 5, Figure S4A, B), thus providing a superior alternative to the EP model of Belfroid et al.^22^ for risk assessment.

The Belfroid et al.^22^ model consistently overestimated internal concentrations of *L. terrestris* by up to a factor of 35. This is most likely because the model parameters including uptake efficiency and feeding rate were calibrated for the relatively smaller earthworm *E. andrei*, and are unlikely to be applicable to *L. terrestris*. Feeding rate can vary over at least 2 orders of magnitude and varies with substrate and species.^38^ As far as we are aware there are no studies that determine uptake efficiency for species other than *E. andrei* and the *E. andrei* studies all come from the group of Belfroid et al.^39,40^ These studies indicate that uptake efficiency can vary between one to 2 orders of magnitude. Further study is required to investigate the effect of interspecies variation on uptake efficiency and feeding rate and incorporate this influence into the model. This could improve the performance of the Belfroid et al.^22^ model though we note that our new model already performs well across species.

The Belfroid et al.^22^ model also overestimated internal concentrations of metalaxyl by a factor of up to 19. This herbicide is the most hydrophilic compound in the evaluation data set (log *K*_ow_ 1.99), and is at the bottom of the applicability domain of the Belfroid et al.^22^ model which was calibrated for compounds in the range log *K*_ow_ 2–7. The Belfroid et al.^22^ model assumes two uptake routes, ingestion and uptake from the bulk soil and dermal uptake from the soil solution. Hydrophilic compounds do not partition strongly onto the soil, instead remaining in solution. Consequently, the use of a universal uptake efficiency value for the ingestion route overestimates the importance of this route for compounds that preferentially partition into the soil porewater. Our new model avoids this issue as it is based on regression relationships for overall uptake.

#### 3.3.2 Kinetic Model of Jager et al.^14^

We previously found that of the three existing kinetic models, that of Jager et al.^14^ performed best.^12^ Our current study confirms that this model works fairly well when applied to independent data for PCB 153. When the model was implemented using the parametrized values for all input parameters provided in Jager et al.,^14^ only 56.8% of predicted internal concentrations of PCB 153 in *E. fetida* and *E. andrei* fell within a factor of 3 of measured values (Figure S5C). Both the porewater-based and bulk soil-based models developed in the present study achieved a more accurate prediction, with over 70.5% of the predictions falling within a factor of 3 of the corresponding measured values (Figure S5A, B).

Moreover, the kinetic model of Jager et al.^14^ contains a large number of input parameters to describe separately dermal and intestinal uptake routes for earthworms in soil. These model parameters, such as rate constants for exchange across skin (*k*_s_) and gut wall (*k*_g_) as well as fixed values for feeding process parameters, are chemical and species-dependent, and therefore have to be parametrized for a specific chemical or species of interest prior to making a prediction.^14^ Jager et al.^14^ parametrized their model for tetrachlorobenzene, hexachlorobenzene, and PCB 153, and the earthworm species *E. andrei*. The significant experimental requirements to support calibration limit the applicability and generalizability of the model to nonparameterised chemicals and earthworm species. Furthermore, to obtain dermal uptake data, earthworms were prevented from feeding by glueing their mouths closed; the stress this places upon the earthworms impacts their behavior and may change the efficiency of dermal uptake.^14^ We attempted to use our experimental data to parametrize *k*_s_ and *k*_g_ in Jager et al.’s^14^ model to produce predictive equations for these terms, but this was unsuccessful as the values were co-correlated. By comparison, our new models contain fewer parameters while still having a mechanistic basis, and are applicable across a range of chemicals, soil types, and earthworm species without having to differentiate between various uptake and elimination pathways. Such models require no additional parametrization and exhibit better applicability, generalizability and accessibility, which makes them more practical for application in risk assessment.

## 4 Environmental Policy Implications

The variation of pesticide uptake and elimination rate constants of earthworms was captured by the regression models developed in this study. These models accounted for the key mechanisms involved in bioconcentration by incorporating chemical, soil, and earthworm properties. Our first-order kinetic models were developed using data from five pesticides with a wide range of properties in a wide variety of soils; the models were tested against compounds and soils with a wider range of properties. For both porewater and bulk soil concentrations, our new models displayed a strong and robust capability to predict the uptake of organic compounds by earthworms across pesticides, soils, earthworm species, and various exposure times. This result indicates that differentiating between different uptake and elimination pathways, is unnecessary for predicting bioconcentration in earthworms. In terms of porewater and soil exposure routes, our model was developed using measured values for both porewater and soil concentrations. However, the independent test data used porewater concentrations modeled from soil concentrations. Although our results suggest that it is unnecessary to differentiate between these exposure routes, independent measured porewater concentration data are needed to fully confirm this.

The models presented here were developed using experimental data for nonionizable organic compounds, but they work reasonably well for monovalent ionizable compounds when log *K*_ow_ is replaced with log *D*_ow_ (Figure S6). For complex ionizable substances, including zwitterionic compounds, nonlinear models such as a nonlinear regression model^41^ or a random forest model^42^ have shown a good capability to capture intricate adsorption mechanisms. Therefore, we suggest using these models to predict *K*_d_ when necessary. In addition, our model achieved reasonable predictions for data points outside the applicability domain (NSE > 0.440), indicating that our models have captured the main patterns involved in bioconcentration and possess wider applicability (Figure S7). We recommend that future research efforts expand the training data set to further enhance the model’s generalizability. However, the developed models exclude biotransformation within the earthworm and active excretion as additional elimination pathways, and increased adsorption of the compound to the soil reducing bioavailability. Overestimation of bioaccumulation is likely for the few compounds where these processes are significant. The impact of biotransformation^29,30,33,34^ and changes in adsorption^17,23,24,31^ on bioaccumulation are well documented in the literature. However, information on active excretion is limited.^43^ Based on the fit of the external data to our model, these processes appear to play a minor role for the majority of substances and earthworm species. While any of these processes, if significant, would result in a conservative risk assessment, further work would be useful to optimize model applicability in such circumstances.

Existing EP models, such as those developed by Jager^44^ and Connell and Markwell,^45^ which are recommended by the Technical Guidance Document^9,10^ for risk assessment of secondary poisoning via earthworms perform less well than those developed by Belfroid et al.^12,22^ In this study we show that relative to our new models, the model of Belfroid et al.^22^ performs less well when different earthworm species are considered, and provides less accurate predictions for hydrophilic compounds. Additionally, the kinetic model of Jager et al.^14^ has limited applicability and accessibility for assessing the risks of pesticides to earthworms associated with time-varying exposures due to its complexity. In general, a model should be as complex as needed to explain the available data but no more so. Our new models are functionally simpler than these existing models and do not require additional parametrization, yet they adequately explain the observed data and offer a better predictive capability, all of which support their straightforward implementation into risk assessment frameworks. Furthermore, because earthworms move through the soil during exposure, and exposure levels can vary with depth, risk assessment of pesticides is improved if variable exposure is considered. Because our new model is a kinetic model, it can be linked to behavioral models in which earthworms move through the soil, potentially encountering different concentrations of pesticides, allowing ecological factors to be taken into account in risk assessments.^6,46^ Thus, our models provide an attractive alternative for risk assessment both for a constant exposure concentration and with the potential for application to realistic exposures that may vary in time and space.

## Supporting Information Available

Data set for model training, search terms for the evaluation data set, calculations of RMSE, *Q*_LOO_^2^, CCC and NSE as well as ordinary differential equations, developed regression models, predictive performance of develop models, QMRF (PDF)
Data set for model evaluation (XLSX)
