# Supplementary material for: A User-Friendly Kinetic Model Incorporating Regression Models for Estimating Pesticide Accumulation in Diverse Earthworm Species Across Varied Soils
Source: Environ Sci Technol. 2024 Jul 31;58(32):14555–64. doi: 10.1021/acs.est.4c06642 (PMC11325639; doi:10.1021/acs.est.4c06642)
Supplement: Supplementary file 1 — es4c06642_si_001.pdf [file es4c06642_si_001.pdf]

Supporting Information

A User-Friendly Kinetic Model Incorporating Regression Models for Estimating Pesticide Accumulation in Diverse Earthworm Species Across Varied Soils

Jun Li[1*], Mark E. Hodson[1], Colin D. Brown[1], Melanie J. Bottoms[2], Roman Ashauer[1,3] and Tania Alvarez[2]

[1]Department of Environment and Geography, University of York, York, YO10 5NG, UK
[2]Syngenta Ltd, Jealotts Hill International Research Centre, Warfield, Bracknell, RG42 6EY, UK
[3]Syngenta Crop Protection AG Rosentalstr. 67 4058 Basel Switzerland

* - Corresponding author; Email: jun.li@york.ac.uk

Number of Pages: 26

Number of Figures:7

Number of Tables: 9

1. Training and evaluation datasets

1.1 Dataset for model training

Table S1. Details of dataset for model training (all values were experimentally determined by Li et al.[1] except low K_{ow} and TPSA which were obtained using the ACD/ILab software (v5.0.0.184)).

Species	Compound	Uptake and elimination rate constant				Chemical property				Soil property				Earthworm property		
		log $k_{in,pw}$	log $k_{out,pw}$	log $k_{in,soil}$	log $k_{out,soil}$	log K_{ow}	log K_d	log K_{om}	TPSA	OM	Clay	CEC	pH	lipid	SSA	SSAlipid*
Lumbricus terrestris	lenacil	-1.56	-0.05	-1.30	0.05	1.69	-0.29	1.73	49.4	0.97	4.02	1.41	5.11	1.55	0.70	0.011
	lenacil	-0.73	0.30	-1.09	0.26	1.69	0.26	1.74	49.4	3.36	9.11	6.65	5.65	1.55	0.70	0.011
	lenacil	-1.48	0.02	-1.50	-0.01	1.69	-0.09	1.66	49.4	1.78	10.0	6.83	6.97	1.55	0.70	0.011
	lenacil	-1.04	0.18	-1.52	0.33	1.69	0.54	1.74	49.4	6.40	15.0	16.8	6.22	1.55	0.70	0.011
	lenacil	-0.26	0.58	-1.69	0.45	1.69	1.32	1.72	49.4	39.89	50.0	88.8	6.67	1.55	0.70	0.011
	flutriafol	-1.05	-0.04	-0.97	-0.01	2.30	-0.11	1.90	50.9	0.97	4.02	1.41	5.11	1.55	0.70	0.011
	flutriafol	-0.18	0.14	-1.07	0.14	2.30	0.89	2.37	50.9	3.36	9.11	6.65	5.65	1.55	0.70	0.011
	flutriafol	-0.43	-0.03	-1.00	-0.11	2.30	0.47	2.22	50.9	1.78	10.0	6.83	6.97	1.55	0.70	0.011
	flutriafol	0.08	0.13	-1.00	0.21	2.30	1.12	2.31	50.9	6.40	15.0	16.8	6.22	1.55	0.70	0.011
	flutriafol	0.67	0.36	-1.43	0.29	2.30	1.97	2.37	50.9	39.89	50.0	88.8	6.67	1.55	0.70	0.011
	dieldrin	1.41	-0.92	-0.67	-1.14	3.70	2.00	4.01	12.5	0.97	4.02	1.41	5.11	1.55	0.70	0.011
	dieldrin	1.19	-0.79	-1.03	-0.87	3.70	2.17	3.64	12.5	3.36	9.11	6.65	5.65	1.55	0.70	0.011
	dieldrin	1.61	-1.04	-0.60	-1.11	3.70	2.25	4.00	12.5	1.78	10.0	6.83	6.97	1.55	0.70	0.011
	dieldrin	1.77	-0.58	-0.76	-0.69	3.70	2.45	3.64	12.5	6.40	15.0	16.8	6.22	1.55	0.70	0.011
	dieldrin	1.37	-0.24	-1.37	-0.42	3.70	2.60	3.00	12.5	39.9	50.0	88.8	6.67	1.55	0.70	0.011
	HCB	2.58	-1.09	-0.61	-1.30	5.31	3.14	5.15	0	0.97	4.02	1.41	5.11	1.55	0.70	0.011
	HCB	2.62	-0.83	-0.70	-0.92	5.31	3.26	4.74	0	3.36	9.11	6.65	5.65	1.55	0.70	0.011

Species	Compound	Uptake and elimination rate constant				Chemical property				Soil property				Earthworm property		
		log $k_{in,pw}$	log $k_{out,pw}$	log $k_{in,soil}$	log $k_{out,soil}$	log K_{ow}	log K_d	log K_{om}	TPSA	OM	Clay	CEC	pH	lipid	SSA	SSAlipid*
	HCB	2.45	-1.16	-0.71	-1.34	5.31	3.09	4.84	0	1.78	10.0	6.83	6.97	1.55	0.70	0.011
	HCB	2.80	-0.75	-0.63	-0.84	5.31	3.42	4.62	0	6.40	15.0	16.8	6.22	1.55	0.70	0.011
	HCB	2.53	-0.28	-0.94	-0.25	5.31	3.56	3.96	0	39.9	50.0	88.8	6.67	1.55	0.70	0.011
	p,p'-DDT	0.76	-1.01	-0.71	-1.04	6.63	1.47	3.48	0	0.97	4.02	1.41	5.11	1.55	0.70	0.011
	p,p'-DDT	0.79	-0.81	-1.09	-0.83	6.63	1.88	3.35	0	3.36	9.11	6.65	5.65	1.55	0.70	0.011
	p,p'-DDT	0.77	-1.06	-0.82	-1.13	6.63	1.58	3.33	0	1.78	10.0	6.83	6.97	1.55	0.70	0.011
	p,p'-DDT	1.31	-0.85	-1.02	-0.87	6.63	2.27	3.46	0	6.40	15.0	16.8	6.22	1.55	0.70	0.011
	p,p'-DDT	1.42	-0.29	-1.40	-0.46	6.63	2.58	2.98	0	39.9	50.0	88.8	6.67	1.55	0.70	0.011
Eisenia fetida	lenacil	-0.97	0.42	-0.76	0.43	1.69	-0.28	1.73	49.4	0.97	4.02	1.41	5.11	2.64	1.01	0.027
	lenacil	-0.72	0.24	-0.78	0.55	1.69	0.24	1.72	49.4	3.36	9.11	6.65	5.65	2.64	1.01	0.027
	lenacil	-1.23	0.33	-1.00	0.50	1.69	-0.31	1.44	49.4	1.78	10.0	6.83	6.97	2.64	1.01	0.027
	lenacil	-0.93	0.27	-1.20	0.61	1.69	0.49	1.68	49.4	6.40	15.0	16.8	6.22	2.64	1.01	0.027
	lenacil	-0.44	0.18	-1.43	0.61	1.69	1.39	1.79	49.4	39.9	50.0	88.8	6.67	2.64	1.01	0.027
	flutriafol	-0.53	0.23	-0.58	0.31	2.30	0.07	2.08	50.9	0.97	4.02	1.41	5.11	2.64	1.01	0.027
	flutriafol	0.55	0.58	-0.46	0.50	2.30	0.94	2.41	50.9	3.36	9.11	6.65	5.65	2.64	1.01	0.027
	flutriafol	-0.38	0.18	-0.67	0.33	2.30	0.33	2.08	50.9	1.78	10.0	6.83	6.97	2.64	1.01	0.027
	flutriafol	0.18	0.28	-0.74	0.59	2.30	1.15	2.35	50.9	6.40	15.0	16.8	6.22	2.64	1.01	0.027
	flutriafol	0.42	0.07	-1.19	0.60	2.30	2.04	2.44	50.9	39.9	50.0	88.8	6.67	2.64	1.01	0.027
	dieldrin	1.94	-1.12	-0.11	-1.18	3.70	2.10	4.11	12.5	0.97	4.02	1.41	5.11	2.64	1.01	0.027
	dieldrin	1.97	-0.82	-0.20	-0.71	3.70	2.24	3.72	12.5	3.36	9.11	6.65	5.65	2.64	1.01	0.027
	dieldrin	2.30	-0.93	0.05	-1.04	3.70	2.12	3.87	12.5	1.78	10.0	6.83	6.97	2.64	1.01	0.027
	dieldrin	2.42	-0.59	-0.06	-0.53	3.70	2.46	3.65	12.5	6.40	15.0	16.8	6.22	2.64	1.01	0.027
	dieldrin	1.37	-0.70	-1.21	-0.56	3.70	2.54	2.94	12.5	39.9	50.0	88.8	6.67	2.64	1.01	0.027
	HCB	2.89	-1.14	-0.15	-1.19	5.31	3.21	5.23	0	0.97	4.02	1.41	5.11	2.64	1.01	0.027

Species	Compound	Uptake and elimination rate constant				Chemical property				Soil property				Earthworm property		
		log $k_{in,pw}$	log $k_{out,pw}$	log $k_{in,soil}$	log $k_{out,soil}$	log K_{ow}	log K_d	log K_{om}	TPSA	OM	Clay	CEC	pH	lipid	SSA	SSAlipid*
	HCB	3.10	-0.64	-0.11	-0.74	5.31	3.25	4.72	0	3.36	9.11	6.65	5.65	2.64	1.01	0.027
	HCB	3.09	-0.95	-0.01	-0.98	5.31	3.13	4.87	0	1.78	10.0	6.83	6.97	2.64	1.01	0.027
	HCB	3.48	-0.51	0.05	-0.58	5.31	3.47	4.67	0	6.40	15.0	16.8	6.22	2.64	1.01	0.027
	HCB	2.61	-0.67	-0.87	-0.57	5.31	3.62	4.02	0	39.9	50.0	88.8	6.67	2.64	1.01	0.027
	p,p'-DDT	1.41	-1.28	-0.28	-1.32	6.63	1.68	3.70	0	0.97	4.02	1.41	5.11	2.64	1.01	0.027
	p,p'-DDT	1.33	-1.06	-0.66	-1.12	6.63	1.97	3.44	0	3.36	9.11	6.65	5.65	2.64	1.01	0.027
	p,p'-DDT	1.25	-1.27	-0.40	-1.29	6.63	1.58	3.33	0	1.78	10.0	6.83	6.97	2.64	1.01	0.027
	p,p'-DDT	1.81	-0.90	-0.55	-0.99	6.63	2.29	3.49	0	6.40	15.0	16.8	6.22	2.64	1.01	0.027
	p,p'-DDT	1.46	-0.88	-1.31	-0.99	6.63	2.64	3.04	0	39.9	50.0	88.8	6.67	2.64	1.01	0.027
Aporrectodea caliginosa	lenacil	-1.84	0.08	-1.46	0.14	1.69	-0.67	1.34	49.4	0.97	4.02	1.41	5.11	2.04	1.45	0.029
	lenacil	-0.92	0.26	-0.97	0.44	1.69	0.14	1.61	49.4	3.36	9.11	6.65	5.65	2.04	1.45	0.029
	lenacil	-1.17	0.50	-1.12	0.25	1.69	-0.53	1.22	49.4	1.78	10.0	6.83	6.97	2.04	1.45	0.029
	lenacil	-0.75	0.25	-0.97	0.59	1.69	0.44	1.63	49.4	6.40	15.0	16.8	6.22	2.04	1.45	0.029
	lenacil	-0.03	0.39	-1.08	0.64	1.69	1.26	1.66	49.4	39.9	50.0	88.8	6.67	2.04	1.45	0.029
	flutriafol	-0.64	0.01	-0.55	0.05	2.30	-0.15	1.86	50.9	0.97	4.02	1.41	5.11	2.04	1.45	0.029
	flutriafol	0.32	0.29	-0.51	0.33	2.30	0.84	2.31	50.9	3.36	9.11	6.65	5.65	2.04	1.45	0.029
	flutriafol	0.17	0.24	-0.21	0.23	2.30	0.33	2.08	50.9	1.78	10.0	6.83	6.97	2.04	1.45	0.029
	flutriafol	0.42	0.22	-0.46	0.51	2.30	1.11	2.31	50.9	6.40	15.0	16.8	6.22	2.04	1.45	0.029
	flutriafol	0.60	0.07	-0.86	0.60	2.30	1.87	2.27	50.9	39.9	50.0	88.8	6.67	2.04	1.45	0.029
	dieldrin	1.74	-1.28	-0.31	-1.57	3.70	1.99	4.00	12.5	0.97	4.02	1.41	5.11	2.04	1.45	0.029
	dieldrin	2.41	-0.22	0.13	-0.42	3.70	2.08	3.55	12.5	3.36	9.11	6.65	5.65	2.04	1.45	0.029
	dieldrin	2.42	-0.66	0.36	-0.72	3.70	2.03	3.78	12.5	1.78	10.0	6.83	6.97	2.04	1.45	0.029
	dieldrin	2.68	-0.27	0.16	-0.40	3.70	2.50	3.69	12.5	6.40	15.0	16.8	6.22	2.04	1.45	0.029
	dieldrin	1.80	0.02	-0.67	0.12	3.70	2.53	2.93	12.5	39.9	50.0	88.8	6.67	2.04	1.45	0.029

Species	Compound	Uptake and elimination rate constant				Chemical property				Soil property				Earthworm property		
		log $k_{in,pw}$	log $k_{out,pw}$	log $k_{in,soil}$	log $k_{out,soil}$	log K_{ow}	log K_d	log K_{om}	TPSA	OM	Clay	CEC	pH	lipid	SSA	SSAlipid*
	HCB	2.82	-1.29	-0.26	-1.33	5.31	3.10	5.12	0	0.97	4.02	1.41	5.11	2.04	1.45	0.029
	HCB	3.64	-0.09	0.35	-0.30	5.31	3.19	4.66	0	3.36	9.11	6.65	5.65	2.04	1.45	0.029
	HCB	3.34	-0.54	0.31	-0.65	5.31	3.06	4.81	0	1.78	10.0	6.83	6.97	2.04	1.45	0.029
	HCB	3.61	-0.26	0.17	-0.39	5.31	3.44	4.63	0	6.40	15.0	16.8	6.22	2.04	1.45	0.029
	HCB	2.86	-0.18	-0.81	-0.27	5.31	3.56	3.95	0	39.9	50.0	88.8	6.67	2.04	1.45	0.029
	p,p'-DDT	0.95	-1.66	-0.56	-1.66	6.63	1.41	3.42	0	0.97	4.02	1.41	5.11	2.04	1.45	0.029
	p,p'-DDT	1.29	-0.71	-0.57	-0.80	6.63	1.70	3.17	0	3.36	9.11	6.65	5.65	2.04	1.45	0.029
	p,p'-DDT	1.01	-1.31	-0.54	-1.36	6.63	1.54	3.29	0	1.78	10.0	6.83	6.97	2.04	1.45	0.029
	p,p'-DDT	1.65	-0.90	-0.70	-0.91	6.63	2.29	3.48	0	6.40	15.0	16.8	6.22	2.04	1.45	0.029
	p,p'-DDT	1.12	-0.77	-1.48	-0.74	6.63	2.49	2.89	0	39.9	50.0	88.8	6.67	2.04	1.45	0.029

HCB is hexachlorobenzene; log $k_{in,soil}$, log $k_{in,pw}$, log $k_{out,soil}$, log $k_{out,pw}$ are the logarithmic transformation of the uptake rate constants in tissue from soil (kg soil kg^{-1} earthworm d^{-1}), porewater (L porewater kg^{-1} earthworm d^{-1}) and, elimination rate constants (d^{-1}) calculated based on bulk soil and porewater concentrations, respectively. log K_{ow}, and TPSA are the octanol-water partition coefficient and fragment−based polar surface area from N, O, S, P polar coefficients, respectively, calculated by the ACD/ILab software (v5.0.0.184); log K_d and log K_{om} are the logarithmic transformation of the experimentally determined sorption coefficient calculated by the ratio of chemical concentration in soil to concentration in porewater and experimentally determined sorption coefficient normalised to organic matter content, respectively; OM, Clay, CEC, and pH are soil organic matter content (%), clay content (%), cation exchange capacity (cmol+/kg), and soil pH, respectively; lipid, SSA, and SSAlipid are earthworm lipid content (%), specific surface areas (m^2 kg^{-1}), and earthworm specific surface area multiplied by earthworm lipid content, respectively.

1.2 Search terms used for generating evaluation datasets

To find earthworm accumulation data in the literature Google scholar, Web of Science, and Science Direct were searched over the period 2002 to 2023 using the following search terms:

"Bioaccumulation"
"Bioconcentration"
"Uptake"
"Bioavailability"

Each of these were combined with
"organic chemicals in earthworms"
"pesticides in earthworms"
"pharmaceuticals in earthworms"

2.Calculations of RMSE, Q^2_{LOO}, CCC and NSE as well as ordinary differential equations

The root-mean squared error (RMSE), leave-one-out cross-validated correlation coefficient (Q^2_{LOO}), leave-one-out cross-validated concordance correlation coefficient (CCC) and Nash−Sutcliffe Efficiency (NSE) were calculated using the following equations (Equations. S1, S2, S3 and S4):

$$RMSE = \sqrt{\frac{\sum_{i=1}^{n}\left(Y_i^{Obs} - Y_i^{Pred}\right)^2}{n}} \quad \text{(S1)}$$

$$Q_{LOO}^2 = 1 - \left[\frac{\sum_{i=1}^{n}\left(Y_i^{Obs} - Y_i^{Pred}\right)^2}{\sum_{i=1}^{n}\left(Y_i^{Obs} - Y^{Obs,\ Mean}\right)^2}\right] \quad \text{(S2)}$$

$$CCC = \frac{2 \cdot cov\left(Y_i^{Obs}, Y_i^{Pred}\right)}{\sigma_{Y_i^{Obs}}^2 + \sigma_{Y_i^{Pred}}^2 + \left(Y^{Obs,\ Mean} - Y^{Pred,\ Mean}\right)^2} \quad \text{(S3)}$$

$$NSE = 1 - \left[\frac{\sum_{i=1}^{n}(Y_i^{Obs} - Y_i^{Pred})^2}{\sum_{i=1}^{n}(Y_i^{Obs} - Y^{Obs,\ Mean})^2}\right] \quad \text{(S4)}$$

where Y_i^{Obs} and Y_i^{Pred} are the *i*th observed and predicted values, respectively. $Y^{Obs,\ Mean}$ and $Y^{Pred,\ Mean}$ are the average of the observed and predicted values, respectively. n is the number of observations. $cov(Y_i^{Obs}, Y_i^{Pred})$ is the covariance between the observed and predicted values. $\sigma^2_{Y_i^{Obs}}$ and $\sigma^2_{Y_i^{Pred}}$ are the variance of the observed values, respectively.

The ordinary differential equations (S5 and S6) for Equations 1 and 4 provided in the manuscript and implemented in Matlab are:

$$\frac{dC_{earthworm}}{dt} = k_{in} \cdot C - k_{out} \cdot C_{earthworm} \quad \text{(S5)}$$

$$\frac{dC}{dt} = -k_0 \cdot C \quad \text{(S6)}$$

where *C* is the concentration of substance in either the porewater (mg L^{-1}) or bulk soil (mg kg^{-1} dry weight); k_0 is the degradation rate constant (d^{-1}) calculated based on chemical concentrations in porewater or bulk soil; $C_{earthworm}$ is the concentration of substance in the earthworm (mg kg^{-1} wet weight); k_{in} is the uptake constant for tissue related to either porewater (L porewater kg^{-1} earthworm d^{-1}) or soil (kg soil kg^{-1} earthworm d^{-1}) concentrations, k_{out} is the elimination rate constant (d^{-1}); *t* is time since initial exposure (d).

3. Regression models for predicting uptake rate constant (k_{in}) and elimination rate constant (k_{out})

Table S6. Multiple linear regression models for predicting porewater-based k_{in} (log $k_{in,pw}$)

Model Summary									
Model	R	R Square	Adjusted R Square	Std. Error of the Estimate	Change Statistics				
					R Square Change	F Change	df1	df2	Sig. F Change
1	0.942[a]	0.886	0.885	0.4908449	0.886	569.829	1	73	0.000
2	0.971[b]	0.943	0.941	0.3501824	0.057	71.424	1	72	0.000
3	0.982[c]	0.964	0.962	0.2815353	0.021	40.392	1	71	0.000
Model 1. Predictors: (Constant), log K_{om}									
Model 2. Predictors: (Constant), log K_{om}, log OM									
Model 3. Predictors: (Constant), log K_{om}, log OM, log SSAlipid									
Dependent Variable: log $k_{in,pw}$									

Table S7. Multiple linear regression models for predicting porewater-based k_{out} (log $k_{out,pw}$)

Model Summary									
Model	R	R Square	Adjusted R Square	Std. Error of the Estimate	Change Statistics				
					R Square Change	F Change	df1	df2	Sig. F Change
1	.850[a]	0.723	0.719	0.3103955	0.723	190.294	1	73	0.000
2	.897[b]	0.805	0.800	0.2620042	0.082	30.456	1	72	0.000
Model 1. Predictors: (Constant), TPSA									
Model 2. Predictors: (Constant), TPSA, log OM									
Dependent Variable: log $k_{out,pw}$									

Table S8. Multiple linear regression models for predicting bulk-soil-based k_{in} (log $k_{in,soil}$)

Model Summary									
				Std. Error	Change Statistics				
Model	R	R Square	Adjusted R Square	of the Estimate	R Square Change	F Change	df1	df2	Sig. F Change
1	.629[a]	0.396	0.387	0.3907500	0.396	47.776	1	73	0.000
2	.787[b]	0.620	0.609	0.3120737	0.224	42.448	1	72	0.000
3	.859[c]	0.738	0.727	0.2606332	0.119	32.226	1	71	0.000
Model 1. Predictors: (Constant), log K_{om}									
Model 2. Predictors: (Constant), log K_{om}, log SSAlipid									
Model 3. Predictors: (Constant), log K_{om}, log SSAlipid, log OM									
Dependent Variable: log $k_{in,soil}$									

Table S9. Multiple linear regression models for predicting bulk-soil-based k_{out} (log $k_{out,soil}$)

Model Summary									
				Std. Error	Change Statistics				
Model	R	R Square	Adjusted R Square	of the Estimate	R Square Change	F Change	df1	df2	Sig. F Change
1	.873[a]	0.763	0.759	0.3316837	0.763	234.670	1	73	0.000
2	.933[b]	0.871	0.867	0.2466628	0.108	59.997	1	72	0.000
3	.938[c]	0.880	0.875	0.2389334	0.010	5.734	1	71	0.019
Model 1. Predictors: (Constant), TPSA									
Model 2. Predictors: (Constant), TPSA, log OM									
Model 3. Predictors: (Constant), TPSA, log OM, log SSAlipid									
Dependent Variable: log $k_{out,soil}$									

4. Evaluation of the predictive performance of our new kinetic model based on bulk-soil concentrations against the independent data for phenanthrene, pyrene, and other organic compounds

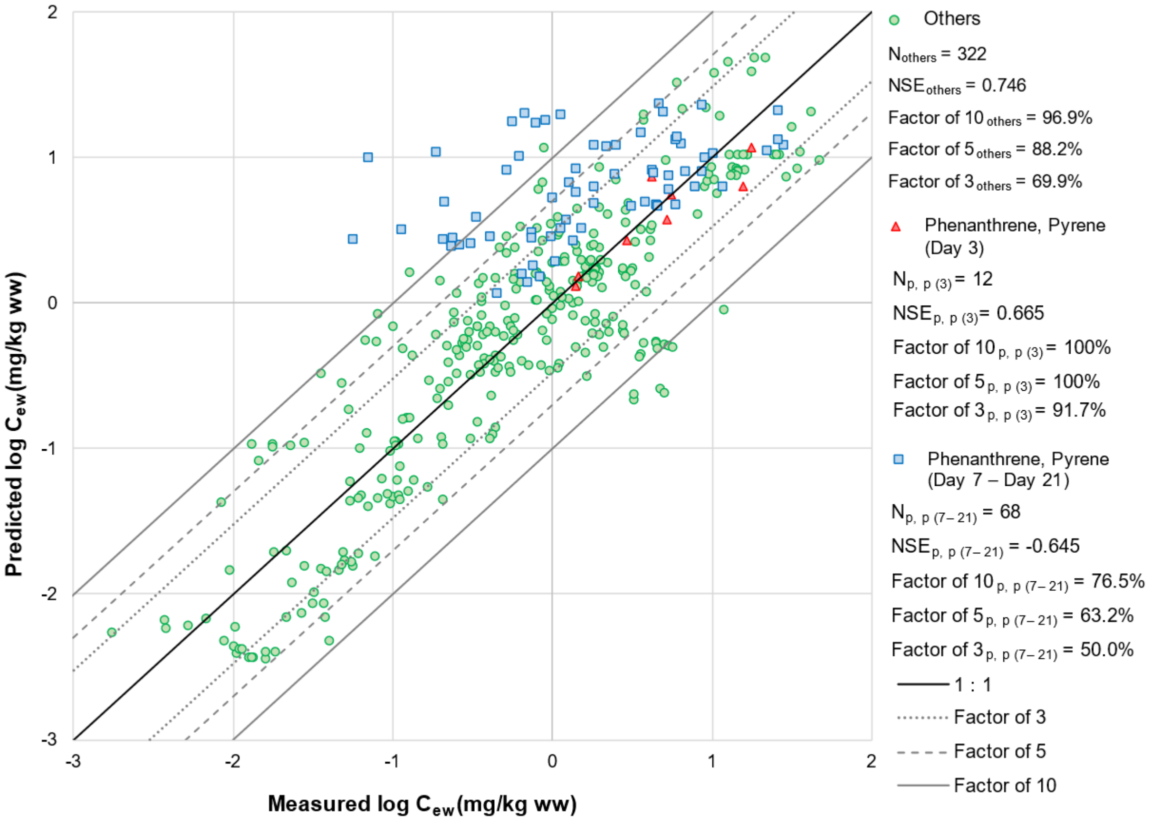

Figure S1. Evaluation of the predictive performance of our new kinetic model based on bulk-soil concentrations against the independent data for phenanthrene, pyrene, and other organic compounds. 'Others' (in green) is the full evaluation dataset excluding data for phenanthrene and pyrene. "p,p (3)" and "p,p (7 − 21)" and "Phenanthrene, Pyrene (Day 3)" and "Phenanthrene, Pyrene (Day 7 – Day 21)" are the evaluation datasets for phenanthrene and pyrene measured in the uptake phase at Day 3 (red triangles) and for Days 7 to 21 (blue squares), respectively. The central black solid line represents a perfect model fit (1:1 line). The grey dotted, grey dashed, and outer solid lines represent a three-fold, five-fold, and ten-fold difference between the predicted and observed values, respectively.

5. Evaluation of the predictive performance of our new kinetic model based on bulk-soil concentrations against the independent data at different exposure time periods

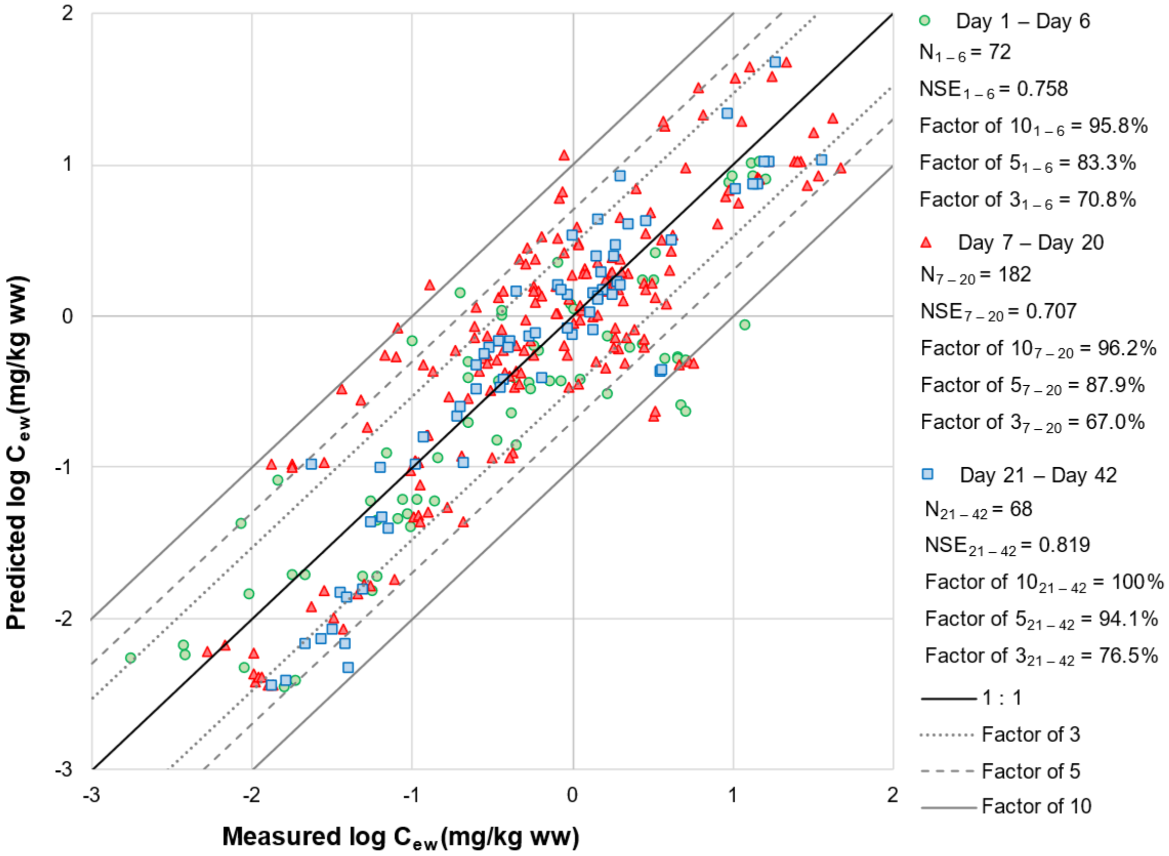

Figure S2. Evaluation of the predictive performance of our new kinetic model based on bulk-soil concentrations against the independent data at different exposure time periods. Data for phenanthrene and pyrene are excluded. “1 − 6”, “7 − 20”, and “21 − 42” and Day 1 – Day 6, Day 7 – Day 20, and Day 21 – Day 42 are the subsets of the evaluation dataset for uptake from Days 1 to 6 (green circles), Days 7 to 20 (red triangles), and Days 21 to 42 (blue squares), respectively. The central black solid line represents a perfect model fit (1:1 line). The grey dotted, grey dashed, and outer solid lines represent a three-fold, five-fold, and ten-fold difference between the predicted and observed values, respectively.

6. Evaluation of the predictive performance of our new kinetic model based on bulk-soil concentrations against the independent data for different earthworm species

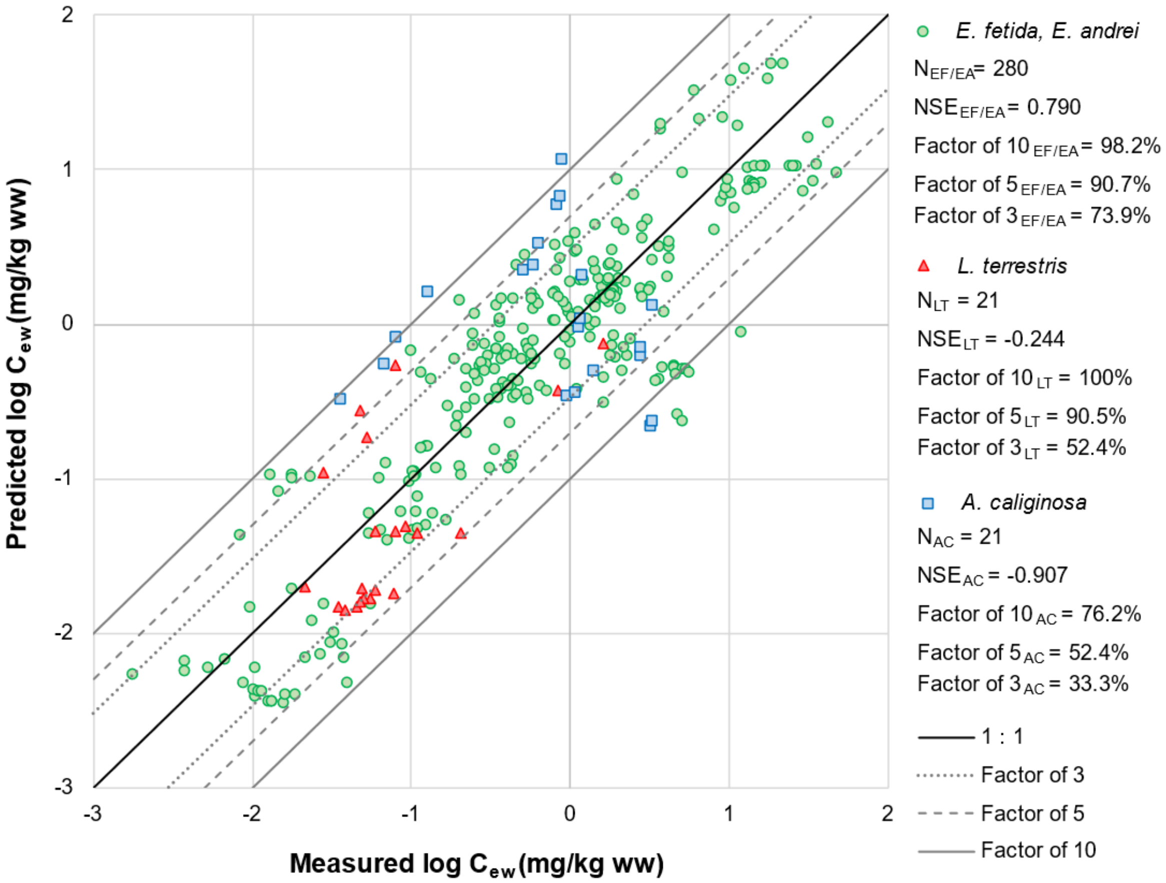

Figure S3. Evaluation of the predictive performance of our new kinetic model based on bulk-soil concentrations against the independent data for different earthworm species. Data for phenanthrene and pyrene are excluded. “EF/EA” (green circles), “LT” (red triangles), and “AC” (blue squares) represent data for the earthworm species *E. fetida, E. andrei*, *L. terrestris,* and *A. caliginosa,* respectively. The central black solid line represents a perfect model fit (1:1 line). The grey dotted, grey dashed, and outer solid lines represent a three-fold, five-fold, and ten-fold difference between the predicted and observed values, respectively.

7. Comparison of the predictive performance of our new models and the existing EP model against data for steady-state or maximum internal concentrations

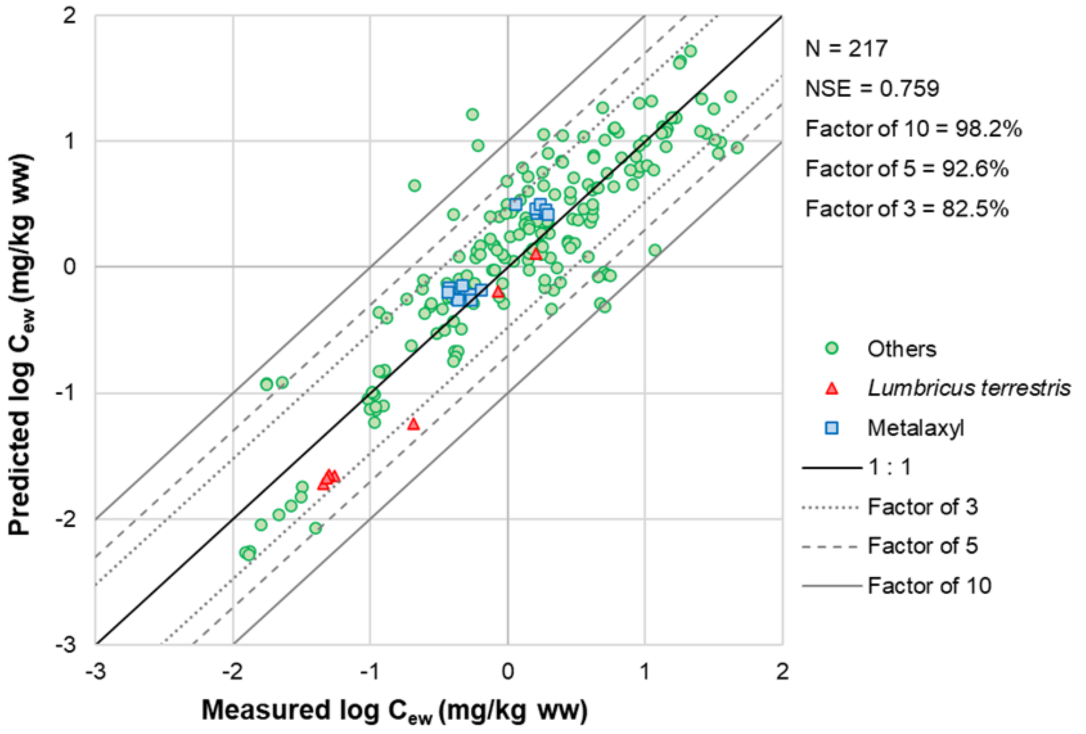

(A)

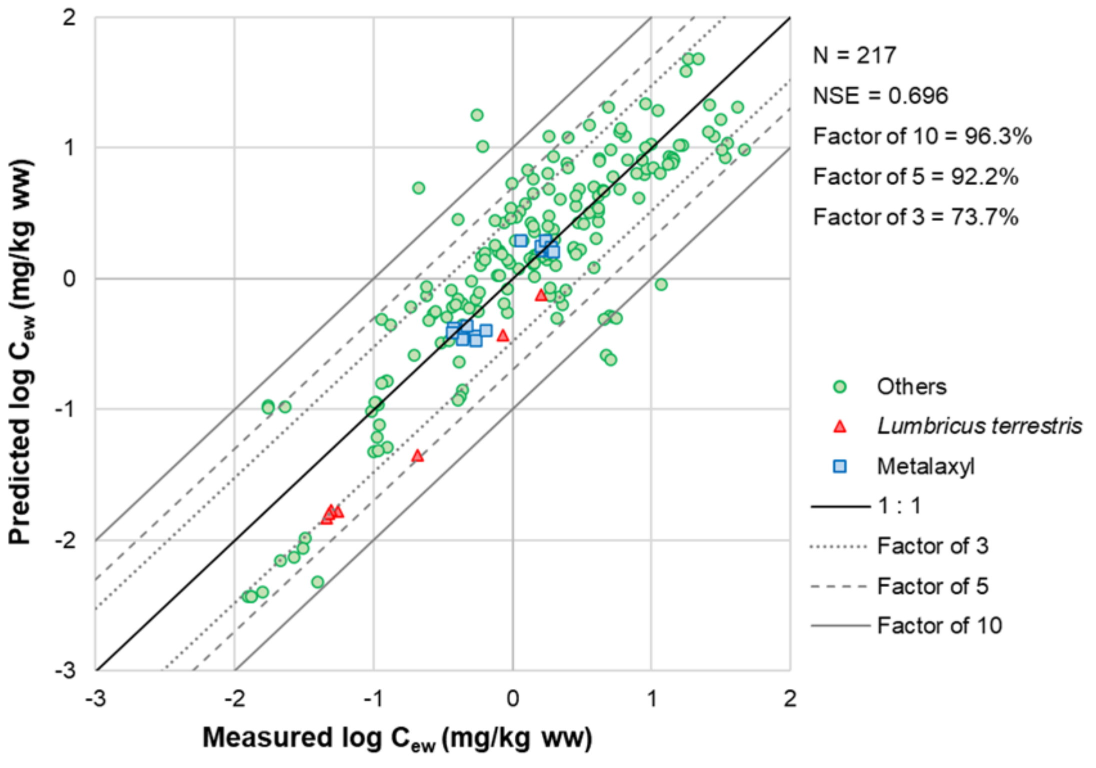

(B)

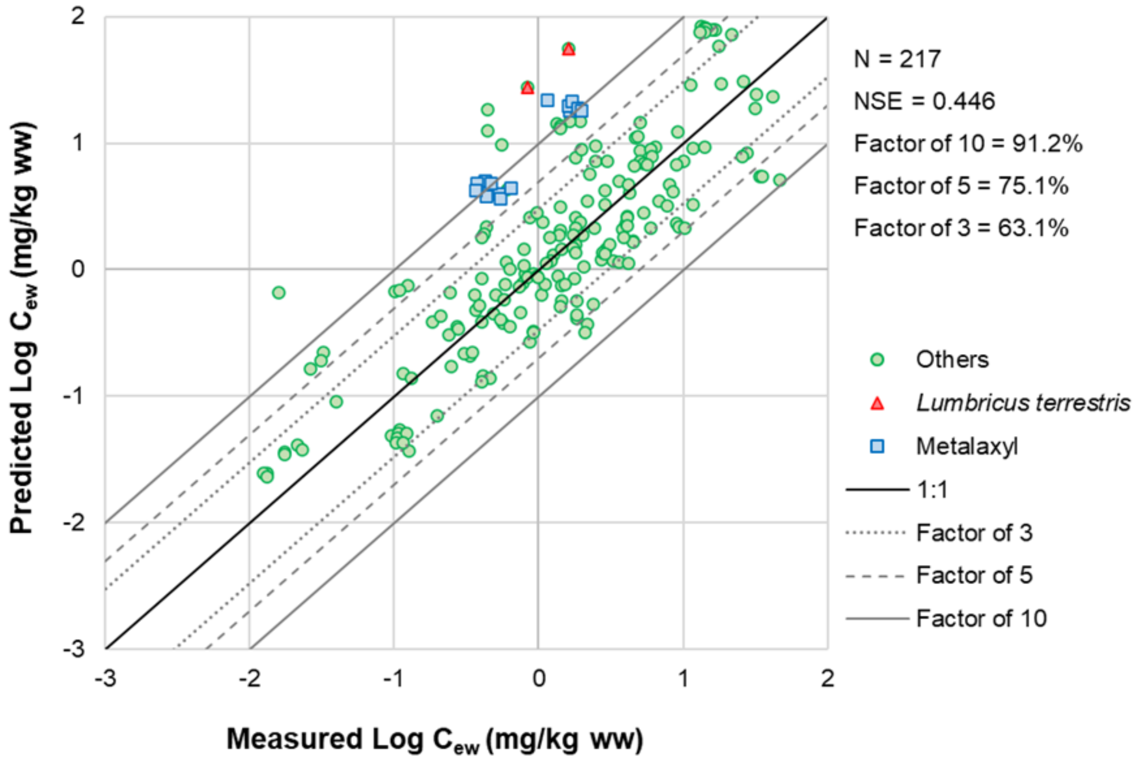

(C)

Figure S4. Predicted steady-state or maximum internal concentrations in earthworms determined using: A) porewater concentrations from the independent dataset based on our newly developed kinetic model; B) bulk-soil concentrations from the independent dataset based on our newly developed kinetic model; and C) the EP model of Belfroid et al.[22]. Others (green circles) represents the independent data excluding data for *Lumbricus terrestris* (red triangles) and metalaxyl (blue squares). The central black solid line represents a perfect model fit (1:1 line). The grey dotted, grey dashed, and outer solid lines represent a three-fold, five-fold, and ten-fold difference between the predicted and observed values, respectively.

8. Comparison of the predictive performance of our new kinetic models and the existing kinetic model against the independent data for PCB 153

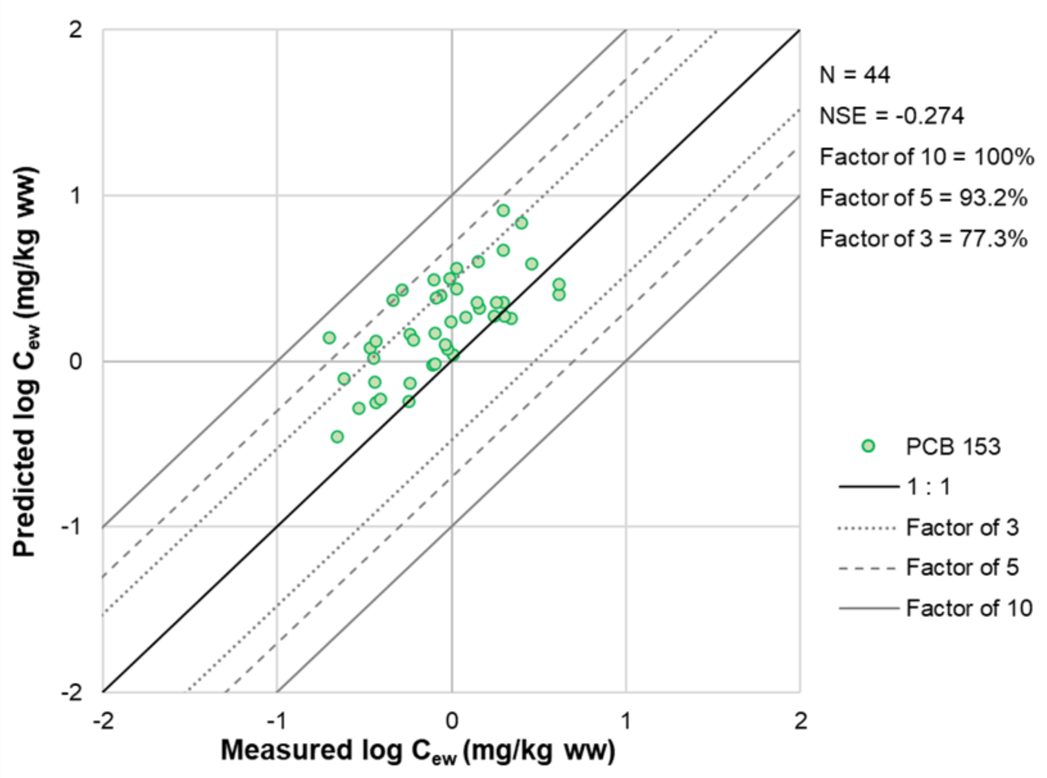

Predicted log C_{ew} (mg/kg ww)
Measured log C_{ew} (mg/kg ww)
N = 44
NSE = -0.274
Factor of 10 = 100%
Factor of 5 = 93.2%
Factor of 3 = 77.3%
PCB 153
1 : 1
Factor of 3
Factor of 5
Factor of 10

(A)

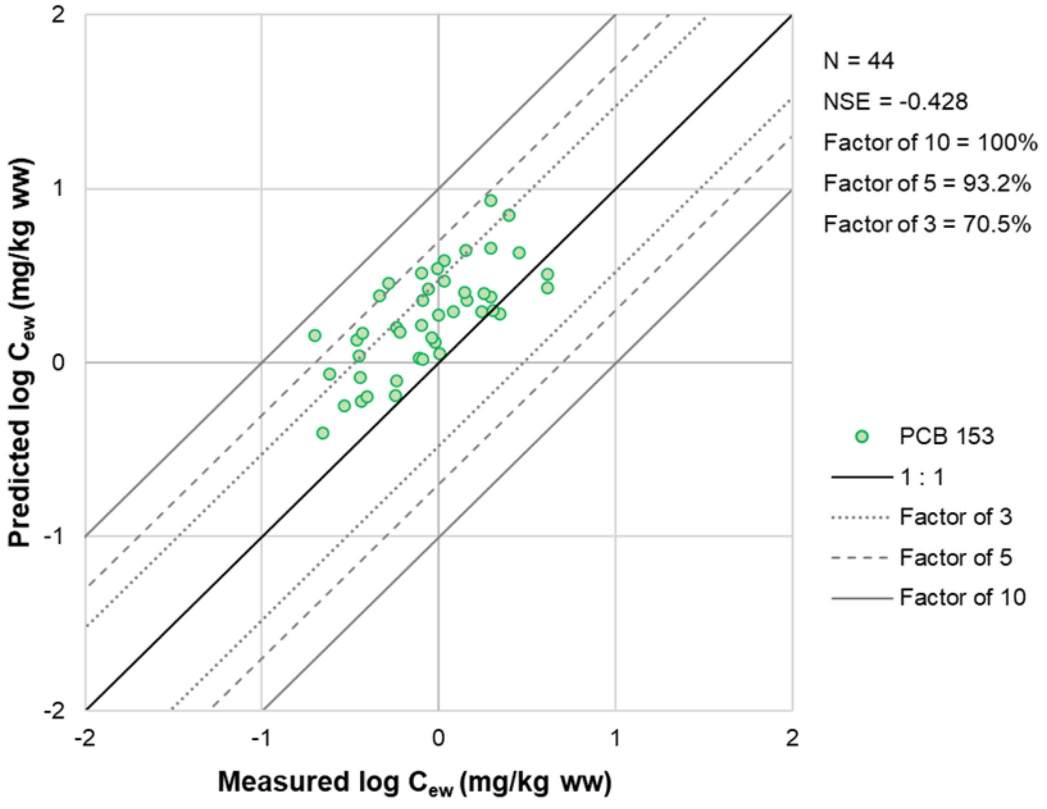

Predicted log C_{ew} (mg/kg ww)
Measured log C_{ew} (mg/kg ww)
N = 44
NSE = -0.428
Factor of 10 = 100%
Factor of 5 = 93.2%
Factor of 3 = 70.5%
PCB 153
1 : 1
Factor of 3
Factor of 5
Factor of 10

(B)

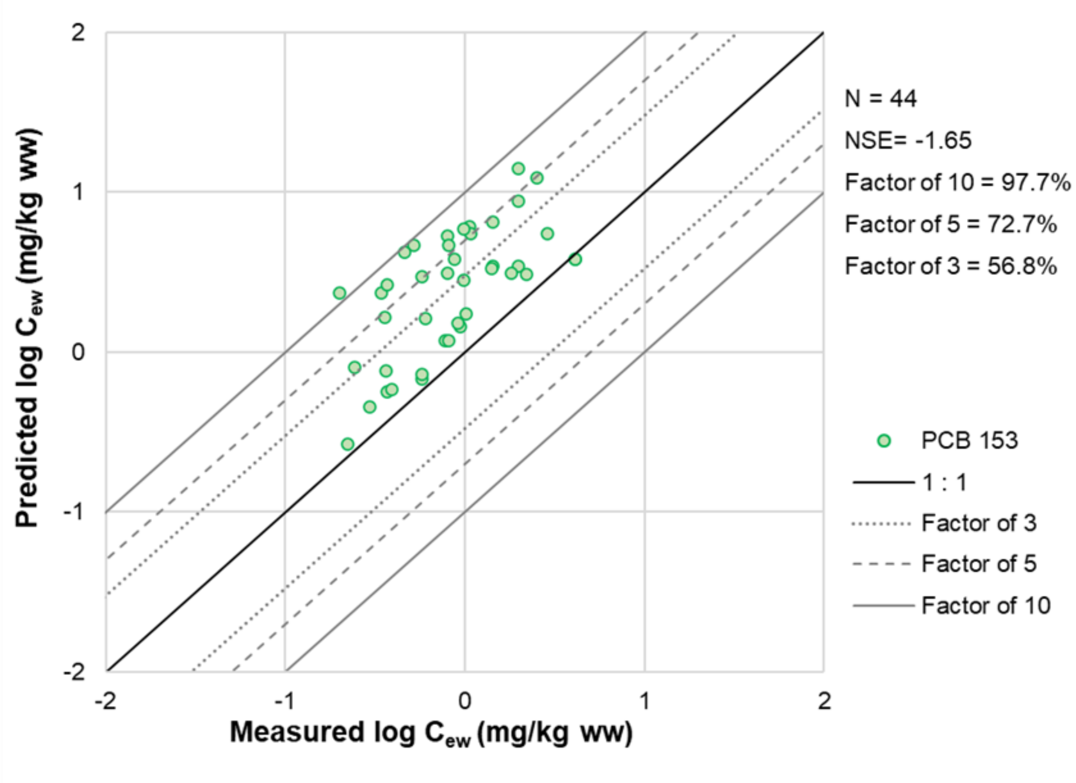

(C)

Figure S5. Comparison of the predictive performance of our new kinetic models based on porewater concentrations (A), bulk-soil concentrations (B), and the kinetic model of Jager et al.[2] (C) against independent data for PCB 153. The central black solid line represents a perfect model fit (1:1 line). The grey dotted, grey dashed, and outer solid lines represent a three-fold, five-fold, and ten-fold difference between the predicted and observed values, respectively.

9. Evaluation of the predictive performance of our new kinetic model based on porewater and bulk-soil concentrations against the independent data for non-ionisable and ionisable compounds

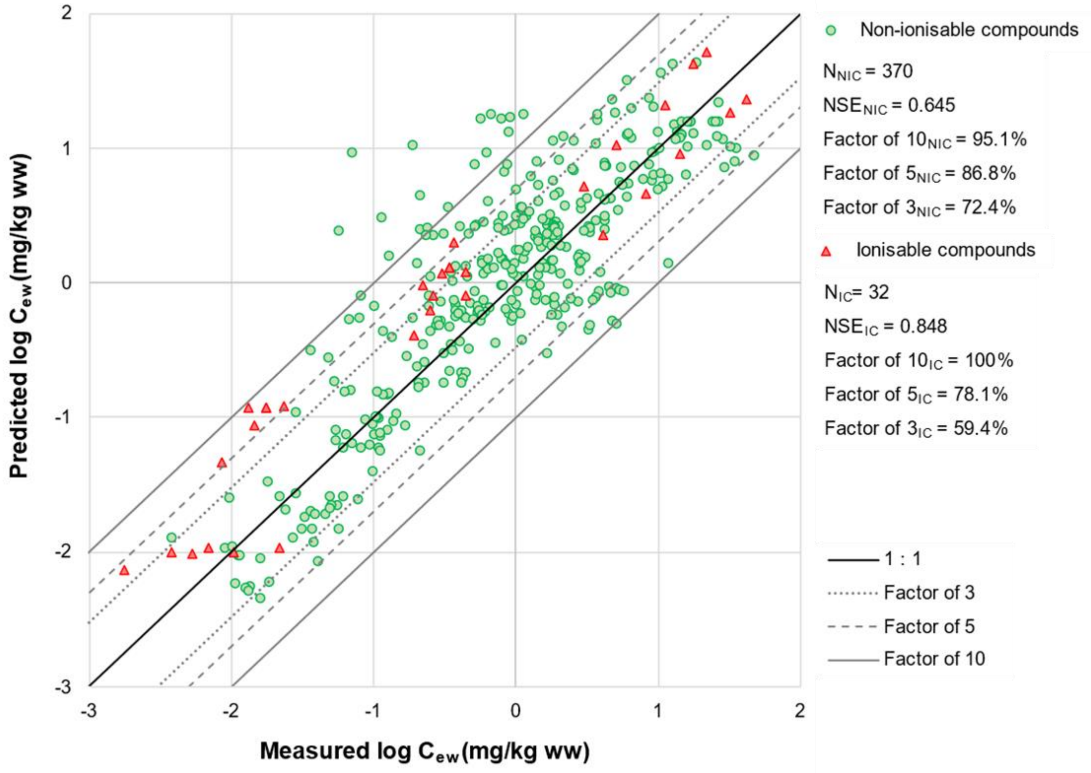

(A)

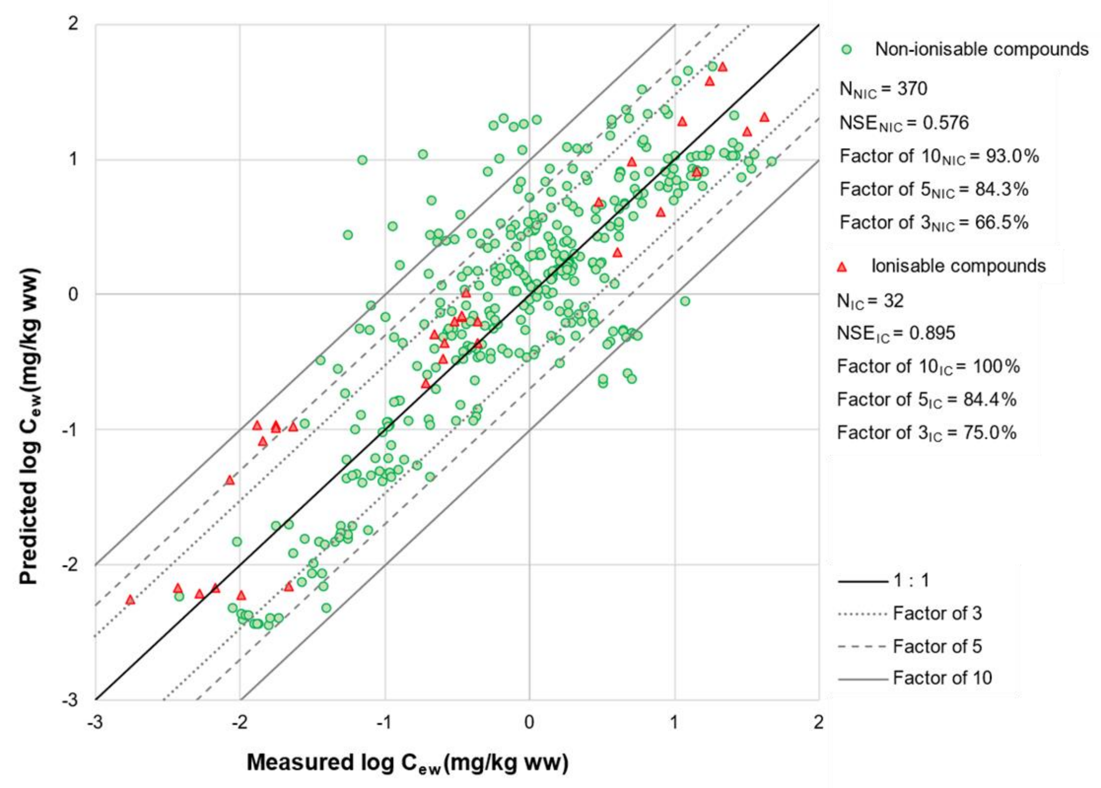

(B)

Figure S6. Evaluation of the predictive performance of our new kinetic model based on porewater (A) and bulk-soil (B) concentrations against the independent data for non-ionisable and ionisable compounds. “NIC” (green circles) and “IC” (red triangles) represent data for non-ionisable and ionisable compounds, respectively. The central black solid line represents a perfect model fit (1:1 line). The grey dotted, grey dashed, and outer solid lines represent a three-fold, five-fold, and ten-fold difference between the predicted and observed values, respectively.

10. Evaluation of the predictive performance of our new kinetic model based on porewater and bulk-soil concentrations against the independent data both within and outside the applicability domain of the models

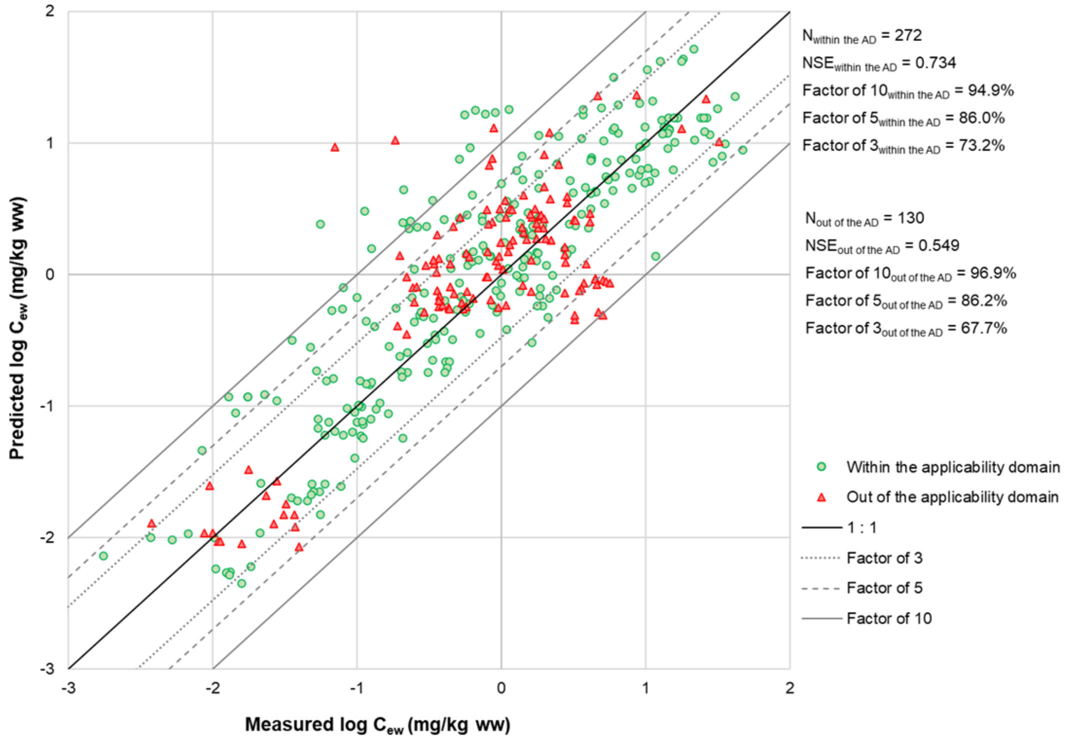

(A)

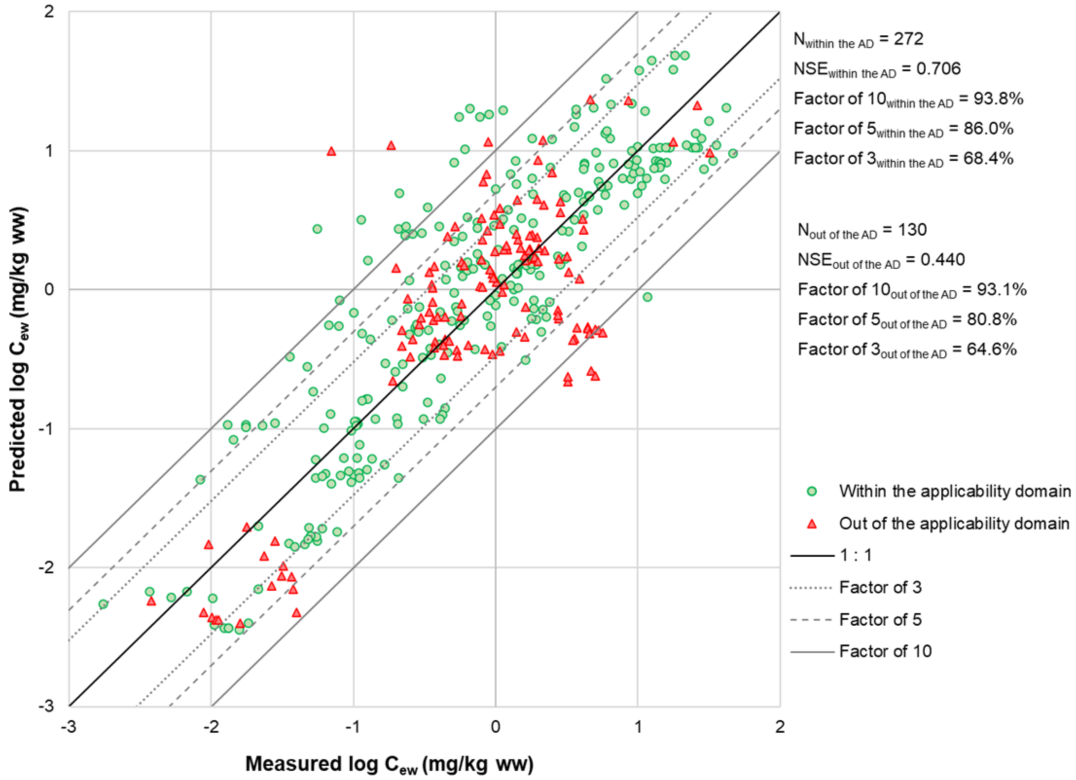

(B)

Figure S7. Evaluation of the predictive performance of our new kinetic model based on porewater (A) and bulk-soil (B) concentrations against the independent data both within and outside the applicability domain of the models. “within the AD” (green circles) and “out of the AD” (red triangles) represent data within and outside the applicability domain of the developed models, respectively. The central black solid line represents a perfect model fit (1:1 line). The grey dotted, grey dashed, and outer solid lines represent a three-fold, five-fold, and ten-fold difference between the predicted and observed values, respectively.

Reference

(1) Li, J.; Hodson, M.E.; Brown, C.D.; Bottoms, M.J.; Ashauer, R.; Alvarez, T. Earthworm lipid content and size help account for differences in pesticide bioconcentration between species. *J. Hazard. Mater.* **2024**, *468*, 133744.

(2) Jager, T.; Fleuren, R.H.; Hogendoorn, E.A.; De Korte, G. Elucidating the routes of exposure for organic chemicals in the earthworm, *Eisenia andrei* (Oligochaeta). *Environ. Sci. Technol.* **2003**, *37*, 3399-3404.

11. QSAR Model Reporting Formats

1. QSAR Identifier

1.1. QSAR identifier (title): Predictive Models for Uptake (k_{in}) and Elimination (k_{out}) Rate Constants of Pesticides in Earthworms

1.2. Other related models: Not specified

1.3. Software coding the model:

- SPSS (version 25.0) for multiple-linear regression analysis
- MATLAB (R2021b) with the BYOM modelling platform (version 6.0)
- R software (R version 3.5.1) for internal validation calculations (Q^2_{LOO} and CCC)

2. General Information

2.1. Date of QMRF: 20/06/2024

2.2. QMRF author(s) and contact details:

- Jun Li, University of York, Department of Environment and Geography, York, YO10 5NG, UK; jun.li@york.ac.uk
- Mark E. Hodson, University of York, Department of Environment and Geography, York, YO10 5NG, UK; mark.hodson@york.ac.uk
- Colin D. Brown, University of York, Department of Environment and Geography, York, YO10 5NG, UK; colin.brown@york.ac.uk
- Melanie J. Bottoms, Syngenta Ltd, Jealotts Hill International Research Centre, Warfield, Bracknell, RG42 6EY, UK; melanie.bottoms@syngenta.com
- Roman Ashauer, Syngenta Crop Protection AG Rosentalstr. 67 4058 Basel Switzerland; roman.ashauer@syngenta.com
- Tania Alvarez, Syngenta Ltd, Jealotts Hill International Research Centre, Warfield, Bracknell, RG42 6EY, UK; Tania.Alvarez@syngenta.com

2.3. Date of QMRF update(s): Not applicable

2.4. QMRF update(s): Not applicable

2.5. Model developer(s) and contact details:

- Jun Li, University of York, Department of Environment and Geography, York, YO10 5NG, UK; jun.li@york.ac.uk

2.6. Date of model development and/or publication: 2024

2.7. Reference(s) to main scientific papers and/or software package:

- Li, J.; Hodson, M. E.; Brown, C. D.; Bottoms, M. J.; Ashauer, R.; Alvarez, T. (unpublished results). A User-Friendly Kinetic Model Incorporating Regression Models for Estimating Pesticide Accumulation in Diverse Earthworm Species Across Varied Soils. *Submitted to Environ. Sci. Technol*.
- Li, J.; Hodson, M.E.; Brown, C.D.; Bottoms, M.J.; Ashauer, R.; Alvarez, T. Earthworm lipid content and size help account for differences in pesticide bioconcentration between species. *J. Hazard. Mater.* **2024**, *468*, 133744.

2.8. Availability of information about the model: Non-proprietary. Defined and available algorithms in the manuscript.

2.9. Availability of another QMRF for exactly the same model: None to date

3. Defining the Endpoint - OECD Principle 1

3.1. Species: Three earthworm species (*Lumbricus terrestris*, *Eisenia fetida*, and *Aporrectodea caliginosa*)

3.2. Endpoint: Uptake (k_{in}) and elimination (k_{out}) rate constants of pesticides in earthworms

3.3. Comment on endpoint: Rate constants measured in both soil porewater and bulk soil concentrations

3.4. Endpoint units:

- k_{in}: L porewater kg^{-1} earthworm d^{-1} or kg soil kg^{-1} earthworm d^{-1}
- k_{out}: d^{-1}

3.5. Dependent variable: log k_{in} and log k_{out}

3.6. Experimental protocol: Values determined following standardized OECD 317 guidelines using data from Li et al.[1] for five pesticides, five soils, and three earthworm species.

3.7. Endpoint data quality and variability: Details are provided in Li et al. (unpublished results).

4. Defining the Algorithm - OECD Principle 2

4.1. Type of model: Multiple linear regression models

4.2. Explicit algorithm:

- For porewater-based k_{in}: log k_{in} = 1.267 * log K_{om} + 0.621 * log *OM* + 1.052 * log *SSAlipid* - 1.506
- For porewater-based k_{out}: log k_{out} = 0.021 * TPSA + 0.301 * log *OM* - 1.057
- For bulk soil-based k_{in}: log k_{in} = 0.266 * log K_{om} + 1.193 * log *SSAlipid* - 0.31 * log *OM* + 0.687
- For bulk soil-based k_{out}: log(k_{out}) = 0.026 * TPSA + 0.397 * log *OM* + 0.336 * log *SSAlipid* - 0.632

4.3. Descriptors in the model:

- log K_{om}: Measured distribution coefficient normalised by soil organic matter
- OM: Soil organic matter content
- SSAlipid: Earthworm specific surface area (SSA) multiplied by lipid content of earthworms (lipid)
- TPSA: Fragment-based polar surface area from N, O, S, P polar coefficients

4.4. Descriptor selection: Stepwise multiple-linear regression with Pearson statistical bivariate correlation to avoid multicollinearity. Additionally, descriptor selection was based on R^2 change and F change in SPSS regression analysis. The optimized descriptors were evaluated based on internal cross-validation, and internal validation calculations (Q^2_{LOO} and CCC) were performed using R software (R version 3.5.1).

4.5. Algorithm and descriptor generation: Implemented in SPSS (version 25.0). Internal validation calculations were conducted using R software (R version 3.5.1).

4.6. Software name and version for descriptor generation: SPSS (version 25.0) and R software (R version 3.5.1) for internal validation calculations

4.7. Chemicals/Descriptors ratio: 75 data points / 3-4 descriptors per model

5. Defining the Applicability Domain - OECD Principle 3

5.1. Description of the applicability domain of the model: Applicable to pesticides with properties within the range of the training dataset: log K_{om} (1.22−5.23), TPSA (0−50.9 $Å^2$), OM (0.97−39.9%), SSA (0.70−1.45 m^2 kg^{-1}), and lipid (1.55−2.64%).

5.2. Method used to assess the applicability domain: Leverage approach with Williams plot for identifying outliers.

5.3. Software name and version for applicability domain assessment: SPSS (version 25.0) for regression and correlation analyses

5.4. Limits of applicability: Defined by the range of chemical and soil properties used in the training dataset.

6. Internal Validation - OECD Principle 4

6.1. Availability of the training set: Yes

6.2. Available information for the training set: All data points and corresponding descriptor values are provided in Li et al*.* (unpublished results).

6.3. Data for each descriptor variable for the training set: All

6.4. Data for the dependent variable for the training set: All

6.5. Other information about the training set: Data obtained from Li et al.[1] and supplemented by experimental data following standardized OECD 317 guidelines.

6.6. Pre-processing of data before modelling: Log transformation of uptake, elimination rate constants, distribution coefficient, soil organic matter content, and earthworm specific surface area (SSA) multiplied by earthworm lipid content (lipid)

6.7. Statistics for goodness-of-fit:

- For porewater-based k_{in}: $R^2 = 0.964$, Adjusted $R^2 = 0.962$, RMSE = 0.276
- For porewater-based k_{out}: $R^2 = 0.805$, Adjusted $R^2 = 0.800$, RMSE = 0.258
- For bulk soil-based k_{in}: $R^2 = 0.738$, Adjusted $R^2 = 0.727$, RMSE = 0.255
- For bulk soil-based k_{out}: $R^2 = 0.880$, Adjusted $R^2 = 0.875$, RMSE = 0.234

6.8. Robustness - Statistics obtained by leave-one-out cross-validation:

- For porewater-based k_{in}: $Q^2_{LOO} = 0.960$, CCC = 0.979
- For porewater-based k_{out}: $Q^2_{LOO} = 0.749$, CCC = 0.858
- For bulk soil-based k_{in}: $Q^2_{LOO} = 0.709$, CCC = 0.833
- For bulk soil-based k_{out}: $Q^2_{LOO} = 0.818$, CCC = 0.901

6.9. Robustness - Statistics obtained by leave-many-out cross-validation: Not specified

6.10. Robustness - Statistics obtained by Y-scrambling: Not specified

6.11. Robustness - Statistics obtained by bootstrap: Not specified

6.12. Robustness - Statistics obtained by other methods: Not specified

6.13. Software used for internal validation: R software (R version 3.5.1) for Q^2_{LOO} and CCC calculations

7. External Validation - OECD Principle 4

7.1. Availability of the external validation set: Yes

7.2. Available information for the external validation set: All data points and corresponding descriptor values are provided in Li et al. (unpublished results).

7.3. Data for each descriptor variable for the external validation set: All

7.4. Data for the dependent variable for the external validation set: All

7.5. Other information about the external validation set: The external validation dataset comprises 402 internal concentration data points for earthworms, collected from 21 studies.

7.6. Experimental design of test set: Data points selected from literature between 2002 and 2022. 402 internal earthworm concentration data points are categorized based on within/outside the applicability domain of the developed models, time series, earthworm species, and steady-state concentrations or maximum concentrations to evaluate the models from different perspectives.

7.7. Predictivity - Statistics obtained by external validation: Predictive performance of the developed models were assessed through Nash−Sutcliffe Efficiencies (NSE).

- NSE = 0.690 for porewater-based predictions
- NSE = 0.643 for bulk soil-based predictions

7.8. Predictivity - Assessment of the external validation set: 95.5% and 93.5% of porewater-based and bulk soil-based predictions, respectively, fall within a factor of 10 of the observed values. The proposed model is predictive for data points outside the applicability domain of the model (NSE > 0.440). Performance for data points within the applicability domain (NSE > 0.706) is slightly better than for those outside it.

7.9. Comments on the external validation of the model: Model performed well for a variety of chemicals, soils, and earthworm species but less accurate for phenanthrene and pyrene due to potential biotransformation of the compound, the initiation of active excretion mechanisms or increased adsorption of the compound to the soil reducing bioavailability.

8. Providing a Mechanistic Interpretation - OECD Principle 5

8.1. Mechanistic basis of the model: Model is based on multiple-linear regression considering chemical, soil, and earthworm properties affecting uptake and elimination rates.

8.2. A priori or a posteriori mechanistic interpretation: A posteriori interpretation provided, indicating log Kom, OM, and SSAlipid as primary factors influencing uptake rates and TPSA and OM influencing elimination rates.

8.3. Other information about the mechanistic interpretation: Not specified

9. Miscellaneous Information

9.1. Comments: Model demonstrates good applicability and predictivity for a wide range of chemicals and earthworm species. Future work could enhance model performance by incorporating biotransformation processes, active elimination and increased adsorption of the compound to the soil reducing bioavailability.

9.2. Bibliography:

- Li, J.; Hodson, M. E.; Brown, C. D.; Bottoms, M. J.; Ashauer, R.; Alvarez, T. (unpublished results). A User-Friendly Kinetic Model Incorporating Regression Models for Estimating Pesticide Accumulation in Diverse Earthworm Species Across Varied Soils. *Submitted to Environ. Sci. Technol*.
- Li, J.; Hodson, M.E.; Brown, C.D.; Bottoms, M.J.; Ashauer, R.; Alvarez, T. Earthworm lipid content and size help account for differences in pesticide bioconcentration between species. *J. Hazard. Mater.* **2024**, *468*, 133744.

9.3. Supporting information: Training and validation datasets, model equations, and detailed statistical analyses are provided in the Li et al. (unpublished results) Supporting Information section.

10. Summary (JRC QSAR Model Database)

10.1. QMRF number: To be assigned

10.2. Publication date: 2024

10.3. Keywords: Uptake rate constant, Elimination rate constant, Pesticides, Earthworms, QSAR, Multiple-linear regression

10.4. Comments: None
